# eHealth Literacy and Its Outcomes Among Postsecondary Students: Systematic Review

**DOI:** 10.2196/64489

**Published:** 2025-07-02

**Authors:** Qin Li, Fang Fang, Yan Zhang, Jiayuan Tu, Pingting Zhu, Lijuan Xi

**Affiliations:** 1 Department of Colorectal Surgery Northern Jiangsu People's Hospital Yangzhou China; 2 Department of Nursing Northern Jiangsu People's Hospital Yangzhou China; 3 Hospital Affiliated Nanjing Medical University (Taizhou People's Hospital) Taizhou China; 4 School of Nursing (School of Public Health) Yangzhou University Yangzhou China; 5 Faculty of Health and Social Sciences Hong Kong Polytechnic University Hong Kong China (Hong Kong)

**Keywords:** eHealth literacy, students, emotions, cognition, behavior

## Abstract

**Background:**

eHealth literacy is essential for postsecondary students; however, few studies have systematically reviewed its levels and related outcomes in this population.

**Objective:**

This study aims to systematically review the existing literature on eHealth literacy levels and the associated outcomes among postsecondary students.

**Methods:**

We systematically searched the PubMed, Web of Science, CINAHL, Embase, Cochrane Library, APA PsycInfo and APA PsycArticles, China National Knowledge Infrastructure, Wanfang Data, Base, and OpenGrey databases for studies published from 2006 to July 01, 2024, following the PRISMA (Preferred Reporting Items for Systematic Reviews and Meta-Analyses) guidelines. Studies were eligible if they were quantitative research papers, assessed eHealth literacy, described the relationship between eHealth literacy and other outcomes, and included postsecondary students. The risk of bias was assessed using the modified Appraisal Tool for Cross-Sectional Studies.

**Results:**

A total of 89 cross-sectional studies were included from among 45,168 eHealth literacy–related publications, with 68 rated as high quality and 21 as moderate quality. Various assessment tools were used across studies, with the eHealth Literacy Scale being the most commonly used (56/89, 63%). Reported eHealth literacy total scores ranged from 23.6 (SD 6.8) to 31.4 (SD 4.4), and mean item scores ranged from 3.42 (SD 0.61) to 4.10 (SD 0.56). Associated outcomes were grouped into cognitive, emotional, and behavioral domains. eHealth literacy was positively associated with cognitive outcomes, including health knowledge, self-efficacy, disease prevention behaviors, and health attitudes. Regarding emotional outcomes, eHealth literacy was linked to higher psychosocial well-being, more positive emotions, and lower negative emotions; however, its associations with overall well-being, depression, and COVID-19 fear were inconclusive. Regarding behavioral outcomes, eHealth literacy was associated with greater use of electronic information, disease prevention practices, volunteerism, and clinical decision-making. Its relationships with health care use, social media engagement, and healthy living were more complex and context-dependent.

**Conclusions:**

eHealth literacy among postsecondary students ranges from moderate-low to moderate-high, with variations due to inconsistent assessment tools. It shows positive associations with cognitive, emotional, and behavioral outcomes, though links to healthy living, digital and health service engagement, and certain psychosocial aspects remain complex. Future research should standardize measurements and explore the mechanisms across disciplines and cultures to guide effective health promotion.

**Trial Registration:**

PROSPERO CRD42024559587; https://www.crd.york.ac.uk/PROSPERO/view/CRD42024559587

## Introduction

Adulthood is recognized as a distinctive developmental phase that marks the critical transition from adolescence to adulthood [1,2]. This period is characterized by significant lifestyle transformations, including independent living, establishing new social networks, and managing personal time and decisions [3]. During this phase, individuals are particularly susceptible to various adverse health behaviors due to factors such as financial stress, academic workload, and inadequate social support [4,5]. Postsecondary students fall precisely within this high-risk period [6].

In addition to facing health risks themselves, postsecondary students play a vital role in public health communication [7]. They often serve as intermediaries between professionals and the broader public, especially when scientific understanding is limited or expert opinions diverge [8,9]. Through academic coursework and faculty interactions, students gain access to professional knowledge, and their daily communication with family and friends connects them closely to their communities [7]. This familiarity with both professional and community perspectives positions them to effectively translate complex health information for a broader audience, making them valuable sources of health information and key actors in health promotion [7].

According to the China Internet Network Information Center, as of December 2024, China had approximately 1.108 billion internet users, with students comprising a significant proportion [10]. The internet provides quick access to a vast amount of up-to-date health information and allows users to interact with health care professionals through platforms such as social media, messaging services, and video streaming sites [11]. Beyond passive information acquisition, the internet supports multidirectional information sharing [12], and many health care providers now use digital platforms to disseminate health knowledge [13]. University students, as active internet users, frequently turn to online sources for health-related information [14].

However, the wide variety and inconsistent quality of online health content pose significant challenges [15]. Students often face challenges in evaluating the credibility and relevance of online health information, increasing the risk of misinformation and biased content shaped by commercial or ideological interests [16]. This underscores the need to assess individuals’ abilities to effectively search for, understand, evaluate, and apply online health information, a concept captured by eHealth literacy [17].

eHealth literacy, introduced by Norman and Skinner in 2006 [18], refers to an individual’s ability to seek, find, understand, and appraise health information from electronic sources and use this knowledge to address health problems. Since its inception, a growing body of literature has sought to refine and expand the measurement of this construct. Several assessment instruments have been developed to operationalize eHealth literacy, including the e-Health Literacy Scale (eHEALS) [19], the eHealth Literacy Scale (EHLS) [20], and the Digital Health Literacy Instrument (DHLI) [21], among others. In addition to instrument development, empirical studies have investigated the levels of eHealth literacy across diverse populations, identified key determinants influencing these levels, and examined the associations between eHealth literacy and a wide range of health outcomes, particularly among healthy adults and individuals with specific medical conditions such as prostate cancer [17,22-25].

Among university students, research has indicated that eHealth literacy is positively associated with lifestyle behaviors [26,27], health information seeking and usage [28], emotional outcomes [29], and other variables. One study has summarized and critically evaluated the levels of eHealth literacy among college students [30]. However, to date, few studies have systematically reviewed the broad range of outcomes associated with eHealth literacy in this population. A comprehensive synthesis of existing findings is therefore urgently needed to better understand these associations and guide future research and practice.

The patient health engagement (PHE) model conceptualizes health engagement as a dynamic process involving the progressive integration of cognitive, emotional, and behavioral components [31]. Rooted in patients’ preferences and lived experiences, it offers a structured framework for designing tailored interventions [32]. According to the model, individuals demonstrate varying engagement levels, with “activation” reflecting gradual advancement across these domains [31]. In this context, Barello et al [33] applied the PHE model and found that eHealth interventions can effectively promote students’ health behavior engagement by targeting these dimensions and supporting incremental change. The model has also been used to examine self-management engagement in individuals with chronic conditions such as diabetes and heart failure [32,34]. Thus, the PHE model might provide a valuable perspective on how eHealth literacy may facilitate behavior change among postsecondary students.

This study aims to conduct a systematic review to synthesize and critically appraise the associations between eHealth literacy, as assessed by various measurement instruments, and a broad range of outcomes among postsecondary students. By providing a comprehensive overview of existing evidence, this review seeks to advance the understanding of the current state of eHealth literacy in this population and its related outcomes and to inform future research in this area.

## Methods

### Review Registration

The review protocol was registered on PROSPERO (International Prospective Register of Systematic Reviews) [35] with identifier number CRD42024559587. We performed this systematic review in accordance with the PRISMA (Preferred Reporting Items for Systematic Reviews and Meta-Analyses) guidelines (Multimedia Appendix 1) [36].

### Data Sources and Search Strategy

A literature search was performed in 10 databases, including PubMed, Web of Science, CINAHL, Embase, Cochrane Library, APA PsycInfo and APA PsycArticles, China National Knowledge Infrastructure, Wanfang Data, Base, and OpenGrey, to identify peer-reviewed publications on eHealth literacy and health outcomes among university students. The search terms involved 2 domains (“eHealth literacy” related and “relate” related). Searches were conducted for publications from January 2006 to July 1, 2024, as the concept of eHealth literacy was first mentioned by Norman and Skinner in 2006 [18]. The detailed search strategy is presented in Multimedia Appendix 2. EndNote and Rayyan were used to support the management of this review.

### Eligibility Criteria

Peer-reviewed empirical studies were screened to assess their relevance to the purpose of this systematic review. Studies were included in our review if they (1) assessed eHealth literacy; (2) described the relationship between eHealth literacy and other outcomes using statistical methods, with reporting of statistically significant associations; and (3) included postsecondary students, such as those in associate degree, vocational, undergraduate, graduate, or PhD programs.

Studies were excluded if they were (1) nonoriginal articles, including reviews, meta-analyses, case reports, editorials, conference abstracts, book chapters, opinion pieces, or letters; and (2) qualitative studies that did not provide quantitative data necessary to examine the relationship between eHealth literacy and relevant outcomes.

### Study Selection

A 2-step selection process was used to identify eligible studies. In the first round, 2 independent investigators (YZ and LX) screened the titles and abstracts of all initially retrieved publications. Next, potentially relevant studies were reviewed in full by the 2 investigators (YZ and LX) to select papers related to our topic. Any discrepancies were resolved by discussion, and a third reviewer (FF) was consulted if necessary.

### Data Collection and Risk of Bias Assessment

For the included studies, data extraction was conducted by 2 investigators (YZ and LX) to collect 3 sets of information: (1) study characteristics, including author, year of publication, country, sample size, and characteristics of the participants (population type, age, and sex); (2) eHealth literacy level and instruments to measure eHealth literacy; and (3) study outcomes and instruments to measure outcomes.

The 2 investigators (YZ and LX) assessed the quality of the eligible publications using the Appraisal Tool for Cross-Sectional Studies (AXIS) [37], which is used for assessing the quality of cross-sectional studies. This tool involves assigning a numerical score to each criterion: 1 point for clear evidence present in papers, and 0 points if absent altogether. The scoring system aligns with previous studies, where a total score of 16 or higher indicates high quality, scores from 12 to 16 indicate moderate quality, and scores below 12 indicate low quality [38].

## Results

### Study Selection

**Figure 1:**
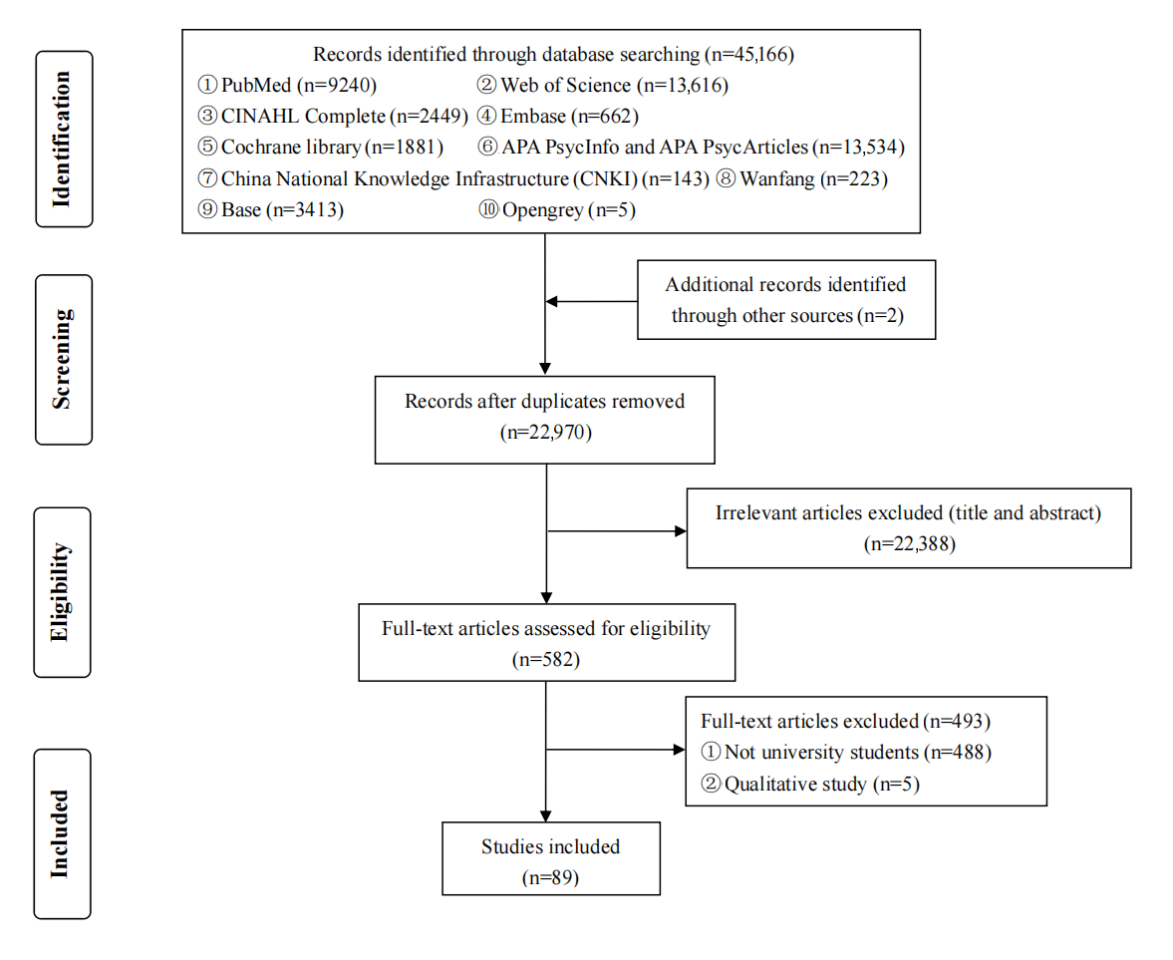
PRISMA (Preferred Reporting Items for Systematic Reviews and Meta-Analyses) flowchart for the study selection process.

A total of 45,168 records were initially identified in electronic databases and imported into SPSS software (IBM Corp). Of these records, 17,131 duplicates were removed from the EndNote database, and 28,037 studies were imported into Rayyan software for title and abstract screening. Following this, 5067 additional duplicates were removed, and 22,388 articles were further removed after the titles and abstracts were found to be irrelevant. Of the 582 publications included for full-text review, 493 articles were excluded for the following reasons: not postsecondary students (n=488) and qualitative study (n=5). A total of 89 articles met the eligibility criteria. The detailed study selection process with the reasons for exclusion during the screening steps is shown in Figure 1.

### Study Characteristics

The overall characteristics of the included studies are summarized in Multimedia Appendix 3. Among the 89 studies, most were from China (n=40), followed by South Korea (n=12), Turkey (n=11), the United States (n=6), Czechia (n=2), Ecuador (n=2), Philippines (n=2), Malaysia (n=2), Austria (n=1), Brazil (n=1), Ghana (n=1), Hungary (n=1), India (n=1), Iran (n=1), Italy (n=1), Japan (n=1), Pakistan (n=1), Romania (n=1), Vietnam (n=1), and both Sweden and Poland (n=1). All retrieved studies were cross-sectional studies using questionnaires. The study participants were grouped into various categories, including associate degree students or vocational students, undergraduate students, graduate students, and PhD students. The sample size in the included studies ranged from 66 [39] to 5641 [40]. Of the 89 studies, 18 (20%) were published from 2014 to 2019, and 71 (80%) were published after 2020.

### Risk of Bias Assessment

The risk of bias assessment revealed that 21 studies were of moderate quality and 68 studies were of high quality according to the AXIS criteria (Multimedia Appendix 4).

### Measurement of eHealth Literacy in the Included Studies

Overall, the 8-item eHEALS was the most frequently used instrument to measure eHealth literacy levels among university students in the included studies (56/89, 63%). Additionally, 5 studies used the modified eHEALS, 6 used the EHLS, 1 used the modified EHLS, 3 used the DHLI, 5 used the modified DHLI, 2 used the DHLI with respect to COVID-19, 1 used COVID-19 Digital Health Literacy, 3 used the eHealth Literacy Scale for College Students, 1 used Lee Sang-rok’s e-Health Literacy Scale, 1 used Perceived e-Health Literacy (PEHL), 1 used eHealth Literacy (EHL), 1 used an e-Health Literacy tool, 1 used the mobile eHealth Literacy Scale (m-eHEALS), 1 used the Self-Developed e-Health Literacy Questionnaire, and 1 used the electronic media health literacy scale.

### eHealth Literacy Levels

Due to the diversity of the instruments used to assess eHealth literacy in the included studies, this review reports the eHealth literacy levels measured by the 3 widely used scales (eHEALS, DHLI, and EHLS).

A total of 56 studies used the eHEALS, which has a total score ranging from 0 to 40 and item scores ranging from 1 to 5. Among these studies, 47 reported eHealth literacy levels. The mean total scores ranged from 23.6 (SD 6.8) to 31.4 (SD 4.4), while the mean item scores ranged from 3.42 (SD 0.61) to 4.10 (SD 0.56). One study reported a median score of 32.00 (IQR 28.00, 2.00), suggesting considerable variation across studies from lower-middle to upper-middle levels.

Three studies used the DHLI, with item scores ranging from 1 to 4, where higher scores indicate higher levels of eHealth literacy. The mean scores ranged from 2.80 (SD 0.42) to 3.10 (SD 0.40), reflecting a moderate level of eHealth literacy among postsecondary students.

Six studies applied the EHLS, of which 3 reported detailed eHealth literacy scores. This scale ranges from 1 (low) to 5 (high). The reported mean scores for functional eHealth literacy ranged from 3.56 (SD 0.77) to 3.94 (SD 0.77), those for interactive eHealth literacy ranged from 3.57 (SD 0.71) to 3.67 (SD 0.67), and those for critical eHealth literacy ranged from 3.59 (SD 0.72) to 3.78 (SD 0.79), indicating moderate to above-moderate levels of eHealth literacy.

### Outcomes and Their Associations With eHealth Literacy

We categorized the reported outcomes using the PHE model, which includes cognitive, emotional, and behavioral components [31]. Among the outcomes identified in our review, behavioral outcomes were the most common (61/89, 69%), followed by cognitive outcomes (34/89, 38%) and emotional outcomes (23/89, 29%).

### Relationship Between eHealth Literacy and Cognitive Outcomes in Postsecondary Students

In terms of cognitive outcomes, eHealth literacy was positively associated with health-related knowledge, including understanding of COVID-19 during the pandemic, infectious diseases, emergency contraception, cervical cancer, human papillomavirus (HPV), and mental health, but had no relationship with COVID-19 vaccination knowledge. Regarding beliefs, higher eHealth literacy was linked to greater self-efficacy, including general self-efficacy, online technology use self-efficacy, and social media use self-efficacy, as well as more positive life perspectives.

In terms of disease-related attitudes, eHealth literacy was negatively associated with misleading disease information (eg, the notion that COVID-19 is a hoax or was artificially created). Conversely, it was positively associated with awareness of disease susceptibility and severity (eg, HPV, cervical cancer, and COVID-19), as well as with favorable attitudes toward COVID-19 prevention and control, COVID-19 vaccination, and vaccination intention during the pandemic. However, there was no relationship between eHealth literacy and the subjective perception of the severity of the pandemic.

Concerning general health attitudes, eHealth literacy was positively related to health perception, risk perception of e-cigarettes, positive attitudes toward healthy nutrition and exercise, intentions for future health maintenance, and willingness to engage in health communication.

Regarding attitudes toward digital health, eHealth literacy was positively associated with the perceived usefulness, satisfaction, trust, enthusiasm, and evaluation of online health information; favorable attitudes toward internet medical advertisements and mobile health software; a proactive approach regarding seeking and using online health information both at present and in the future; and a higher tendency to seek health information. However, it was negatively associated with satisfaction with mobile health software.

Additionally, eHealth literacy was positively associated with attitudes toward the need for volunteer work (Table 1).

**Table 1:** Relationship between eHealth literacy and cognitive outcomes among postsecondary students.

Cognitive outcomes				Relationship^a^
**Knowledge**				
	**Health-related knowledge**			
		**High COVID-19–related knowledge**		
			COVID-19 knowledge	Positive association [19,41]
			COVID-19 vaccination knowledge	No association [42]
		**High knowledge of other diseases**		
			Infectious disease health literacy	Positive association [43]
			Emergency contraception knowledge	Positive association [44]
			Cervical cancer and human papillomavirus knowledge	Positive association [45]
			Mental health literacy	Positive association [46]
**Belief**				
	**Beliefs about self-efficacy**			
		High generalized self-efficacy		Positive association [47,48]
		High online technology use self-efficacy		Positive association [49]
		High social media use self-efficacy		Positive association [50]
	**Beliefs about life**			
		Positive life perspectives		Positive association [20,51]
**Attitude**				
	**Attitudes toward diseases**			
		**Attitudes toward misleading disease information**		
			COVID-19 is a hoax	Negative association [52,53]
			COVID-19 was created	Negative association [52]
		**Perceptions of disease susceptibility and severity**		
			Perceived sensitivity and perceived seriousness of human papillomavirus and cervical cancer	Positive association [45,54]
			COVID-19 would likely be contracted	Positive association [21]
			COVID-19 would severely impact their life	Positive association [21]
			Subjective perception of the severity of the pandemic	No association [21]
		**Positive attitudes toward the prevention and control of diseases**		
			Positive attitudes toward the prevention and control of COVID-19	Positive association [19,48]
			Positive attitudes toward COVID-19 vaccination	Positive association [42]
			COVID-19 vaccination intention	Positive association [21,55,56]
	**Attitudes toward health**			
		Health perception		Positive association [57]
		**Attitudes toward e-cigarettes**		
			E-cigarette risk perception	Positive association [58]
			E-cigarette benefit perception	No association [58]
		Positive attitudes toward healthy nutrition		Positive association [59]
		Positive attitudes toward exercise		Positive association [60]
		Future health maintenance attitudes		Positive association [61]
		Willing to engage in health communication		Positive association [50]
	**Attitudes toward digital tools**			
		**Attitudes toward digital health tools and resources**		
			Satisfaction with mobile health software	Negative association [62]
			Perceived usefulness of online health information	Positive association [39,63]
			Satisfaction with online COVID-19 information	Positive association [64,65]
			Trust in online health information	Positive association [66,67]
			Perceived importance of accessing health resources online	Positive association [39,65]
			Positive attitudes toward internet medical advertisement	Positive association [66]
			Trust in mobile health software	Positive association [62]
			Technology enthusiasm	Positive association [29]
		**Attitudes toward the use of digital health tools and resources**		
			Positive attitudes toward seeking and using online health information now or in the future	Positive association [61,68]
			Health information seeking inclination	Positive association [63]
	**Attitudes toward volunteers**			
		Need for volunteer work		Positive association [69]

^a^The association between eHealth literacy and cognitive outcomes.

### Relationship Between eHealth Literacy and Emotional Outcomes in Postsecondary Students

In terms of emotional outcomes, eHealth literacy was positively associated with psychosocial well-being, including mental health, resistance to peer pressure, and spiritual health, as well as with positive emotional states such as sense of coherence and fulfillment of psychological needs. However, its association with overall well-being remains unclear. eHealth literacy was negatively related to negative emotional outcomes, including anxiety, cyberchondria, and distress arising from online health information seeking. Its relationship with depression and fear of COVID-19 during the pandemic, however, was uncertain (Table 2).

**Table 2:** Relationship between eHealth literacy and emotional outcomes among postsecondary students.

Emotional outcomes			Relationship^a^
**Psychosocial wellness**			
	Mental health		Positive association [70-72]
	Resistance to peer pressure		Positive association [73]
	**Well-being**		Positive association [65,74-76]; No association [77,78]
		Determining the relevance of health information to a personal situation	Positive association
		Searching for online health information	No association
		Generating personal health-related content	No association
		Assessing the credibility of health information	No association [79]
	Spiritual health		Positive association [51]
**Negative emotions**			
	Negative emotions		Negative association [77]
	Depression		Negative association [80]; No association [81]
	**Anxiety**		
		Anxiety	Negative association [81]
		Future anxiety	Negative association [53,64,74,79]
		Health anxiety	Negative association [82]
		Technology anxiety	Negative association [29]
	Fear of COVID-19		Negative association [64,65]; No association [81,83]
	Cyberchondria		Negative association [47,82]
	Distress with online health information seeking		Negative association [84]
**Positive emotions**			
	Sense of coherence (ability to adapt when confronted with adversities or challenges)		Positive association [74,85]
	Satisfaction of psychological needs		Positive association [77]

^a^The association between eHealth literacy and emotional outcomes.

### Relationship Between eHealth Literacy and Behavioral Outcomes in Postsecondary Students

In terms of internet use and health information–seeking behaviors, eHealth literacy was positively associated with health-related social media use, health information seeking (eg, healthy lifestyle and cervical cancer), information processing (eg, accessing eHealth information and detecting online rumors), and effective use of mobile health apps. It was negatively associated with mobile phone addiction. Associations with general social media use and health service use were inconsistent and may vary across eHealth literacy dimensions.

In terms of healthy living, eHealth literacy was positively associated with better physical health. It also showed a positive association with certain domains of healthy lifestyle behaviors, such as maintaining a regular routine, practicing safe sex, and life appreciation. However, the findings were inconsistent regarding the relationship between eHealth literacy and self-care agency, as well as other aspects of healthy lifestyle behaviors, including sleep, diet and nutrition, physical activity, avoidance of harmful substances, interpersonal relationships, health responsibility behaviors, and mental health behaviors.

Regarding disease-related behaviors, eHealth literacy was positively associated with disease prevention behaviors, such as receiving necessary vaccinations, as well as disease management behaviors, including disease coping, dysmenorrhea management, and rational drug use. However, a negative association was found with HPV vaccination. eHealth literacy was also positively linked to certain COVID-19–related behaviors during the pandemic, including handwashing, staying at home except for essential activities, participation in quarantine, and COVID-19 vaccination. However, the findings were inconsistent for behaviors, such as physical distancing and mask-wearing, and no association was found with avoiding crowded places or maintaining regular indoor ventilation.

Additionally, eHealth literacy was positively associated with volunteer behavior and clinical decision-making ability (Table 3).

**Table 3:** Relationship between eHealth literacy and behavioral outcomes among postsecondary students.

Behavioral outcomes			Relationship^a^
**Internet use and health information–seeking behavior**			
	**Social media use**		
		Medical or social media use for health information	Positive association [86]
		Social media use	No association [58]; Positive association [87]
	**Online health information–seeking behavior**		
		Compulsiveness with online health information seeking	No association [84]
		Online health information–seeking behavior	Positive association [28,39,63,84,86]
		Online healthy lifestyle information–seeking behavior	Positive association [61]
	**Health information–seeking behavior**		
		Health information seeking	Positive association [47]
		Actively seeking and obtaining information about cervical cancer	Positive association [54]
	**Information processing**		
		Accessing and using electronic health information	Positive association [67,88]
		Detecting online rumors during public health emergencies	Positive association [89]
	**Usage efficiency and effectiveness of mobile health care apps**		
		Engaging in the efficient use of mobile health care apps	Positive association [90]
		Engaging in the effective use of mobile health care apps	Positive association [90]
	Mobile phone addiction		Negative association [91]
	**Health service use**		
		Making good use of diverse health care services	Positive association for interactive and critical eHealth literacy, and no association for functional eHealth literacy [92]
		Making good use of a multitiered health care system	Positive association for interactive and critical eHealth literacy, and no association for functional eHealth literacy [92]
		Seeking medical advice based on different needs	Positive association for critical eHealth literacy, and no association for functional and interactive eHealth literacy [92]
		Frequency of medical use	Negative association for functional eHealth literacy, positive association for interactive eHealth literacy, and no association for critical eHealth literacy [92]
**Healthy living**			
	Better physical health		Positive association [93]
	Health complaints		No association [78]
	Self-care agency		Positive association [46,73,86]; No association for nonnursing students, and positive association for nursing students [94]
	Healthy lifestyle behavior		Positive association [41,48,51,63,86,95-106]; No association for Koreans, and negative association for Chinese [67]
	Regular routine		Positive association [99-101]
	**Sleep**		
		Staying up late	Negative association [107]
		Obtaining sufficient sleep	Positive association [61,95,108]; No association [26]
	**Diet and nutrition**		
		Nutrition	Positive association [99-102]; No association [51,109]; Positive association for critical eHealth literacy, and no association for functional and interactive eHealth literacy [20]
		Eating breakfast	Positive association [26]
		Balanced dietary behavior	Positive association [61,95,110,111]; Positive association for interactive eHealth literacy, and no association for critical and functional eHealth literacy [112]
		Dietary improvement behavior	Positive association [84]
		Regular eating habits	Positive association for critical eHealth literacy, and no association for functional and interactive eHealth literacy [112]
		Unhealthy food intake	Positive association for critical and functional eHealth literacy, and no association for interactive eHealth literacy [112]
		Healthy consumption pattern	Positive association for interactive and critical eHealth literacy, and no association for functional eHealth literacy [112]
	Physical activity		Positive association [26,61,84,95,99-102,108,110]; No association [51,109,113]; Positive association for critical eHealth literacy, and no association for functional and interactive eHealth literacy [20]
	**Maintaining a lifestyle free of harmful substances**		
		Maintaining a lifestyle free of harmful substances	Positive association [61,99]; No association [100]
		Smoking	Positive association [107,108]; No association [26]
		Alcohol consumption	Positive association [108]; No association [26]
	**Interpersonal relationships**		
		Interpersonal relationships	Positive association [51,61,99-102]; Positive association for functional and critical eHealth literacy, and no association for interactive eHealth literacy [20]
		Online bridging social capital ability	Positive association [87]
		Online bonding social capital ability	No association [87]
	Maintaining safe sex practices		Positive association [61]
	Health responsibility behaviors for maintaining personal and public hygiene		Positive association [99-102]; No association [51]; Positive association for critical eHealth literacy, and no association for functional and interactive eHealth literacy [20]
	Life appreciation behavior		Positive association [99-101]
	**Mental health behavior**		
		Stress management	Positive association [51,99,101,102]; Positive association for critical eHealth literacy, and no association for functional and interactive eHealth literacy [20]
		Promoting mental health behaviors	Positive association [102,106]
		Online psychological help-seeking behavior	Positive association [70]
**Disease-related behavior**			
	**Disease preventive behavior**		
		Get necessary vaccinations	Positive association [61]
		Human papillomavirus vaccination	Negative association [114]
	**COVID‐19–related behavior**		
		COVID‐19–related preventive behavior	Positive association [19,21,40,41,98,115]
		Frequent hand washing	Positive association [81,116]
		Physical distancing	Positive association [81,116]; No association [117]
		Avoiding crowded places	No association [117]
		Wearing a mask	Positive association [81,116]; No association [117]
		Staying at home except for essential activities	Positive association [116]
		Participation in COVID-19 quarantine measures	Positive association [55]
		Regular indoor ventilation	No association [117]
		COVID-19 vaccination behavior	Positive association [42]
	**Disease management behavior**		
		Disease coping behavior	Positive association [118]
		Dysmenorrhea management behavior	Positive association [119]
		Rational drug use	Positive association [120]
**Other behaviors**			
	Volunteer work action		Positive association [69]
	Clinical decision-making ability		Positive association [121]

^a^The association between eHealth literacy and behavioral outcomes.

## Discussion

### Summary of the Review Findings

This systematic review provides a comprehensive examination of eHealth literacy levels and a broad spectrum of associated outcomes among postsecondary students, addressing cognitive, emotional, and behavioral dimensions.

### eHealth Literacy Levels

This review summarizes eHealth literacy levels as assessed by the 3 most widely used instruments (eHEALS, DHLI, and EHLS). Results based on the eHEALS revealed considerable variability, with scores ranging from lower-middle to upper-middle levels. Assessments using the DHLI indicated a moderate level of eHealth literacy among postsecondary students, while findings from the EHLS suggested levels ranging from moderate to above-moderate.

Taken together, these results suggest that postsecondary students generally demonstrate eHealth literacy levels ranging from lower-middle to upper-middle. However, the interpretation is constrained by heterogeneity in measurement tools and scoring systems across studies. Thus, there is a critical need for the adoption of more rigorous and standardized instruments to accurately evaluate eHealth literacy in this population.

### Relationship Between eHealth Literacy and Cognitive Outcomes in Postsecondary Students

Our review demonstrates a positive association between eHealth literacy and general health-related knowledge, including topics such as COVID-19 during the pandemic, infectious diseases, reproductive health, and mental health. This may reflect the ability of individuals with higher eHealth literacy to effectively acquire and apply online health information [122]. However, no significant association was found with COVID-19 vaccination knowledge [42], possibly due to the technical complexity of vaccine-related content, which may exceed the comprehension supported by general eHealth literacy, particularly among nonmedical students [123]. These findings highlight the distinction between general and domain-specific health literacy, suggesting that eHealth literacy alone may be insufficient for understanding complex medical information. Further research is warranted to examine the moderating roles of educational background and targeted interventions in bridging this gap.

Higher eHealth literacy was also linked to greater self-efficacy across general, technological, and social media contexts. This likely results from improved health information access and comprehension, enhancing confidence in managing health issues [124]. Additionally, students with higher eHealth literacy tended to hold more optimistic life views, possibly because access to credible information reduces uncertainty and promotes a positive psychological state [25,79,125]. Their enhanced ability to manage health through information appraisal may strengthen perceived control, thereby promoting self-efficacy and optimism. Nonetheless, further research is needed to clarify these mechanisms [126].

eHealth literacy was associated with more accurate disease-related attitudes. Individuals with higher eHealth literacy exhibited lower acceptance of misinformation and greater awareness of disease susceptibility and severity, likely due to stronger skills in information evaluation and heightened health consciousness [54,127]. Positive attitudes toward COVID-19 prevention and control during the pandemic were positively associated with eHealth literacy. This may reflect the role of adequate health knowledge in shaping attitudes and supporting the adoption of preventive behaviors. Individuals with higher eHealth literacy are more capable of acquiring, evaluating, and applying online health information, which in turn facilitates the development of informed attitudes and corresponding actions [19,128]. However, no significant association was found between eHealth literacy and subjective perceptions of pandemic severity, possibly due to the influence of sensationalized media coverage, which may shape perceptions independently of literacy levels [129].

In terms of general health attitudes, eHealth literacy was positively associated with various outcomes such as health perception, risk perception of e-cigarettes, positive attitudes toward healthy nutrition and exercise, future health maintenance, and willingness to engage in health communication. Individuals with higher eHealth literacy are better able to access, understand, and apply health information, which may enhance their perception of personal health and facilitate the identification of health risks [130-132]. According to the knowledge-attitude-practice (KAP) theory, knowledge forms the foundation of attitudes, suggesting that individuals with higher eHealth literacy are more likely to develop positive health attitudes through active information seeking related to healthy lifestyles [133]. Among nursing undergraduates, higher eHealth literacy appears to enhance the awareness of patients’ health information needs and improve the use of digital tools for information retrieval, thereby strengthening perceived behavioral control and intentions to communicate health information [50]. 

eHealth literacy was positively associated with favorable attitudes toward digital health tools, consistent with the technology acceptance model, which suggests that perceived usefulness and ease of use influence technology adoption [134]. Individuals with higher eHealth literacy are better able to access, understand, and evaluate online health information, likely enhancing their perception of the value and usability of digital tools, thereby fostering greater trust and willingness to use them [63]. Conversely, eHealth literacy was negatively associated with satisfaction with mobile health apps. This may reflect higher expectations and more critical evaluations among individuals with greater eHealth literacy, in contrast to the limited functionality and user experience issues common in many current apps [62,135]. Further research is needed to explore the factors mediating this relationship and to inform user-centered design improvements.

Finally, eHealth literacy was also positively associated with awareness of the need for volunteer engagement. While nursing students generally recognize the importance of volunteering, barriers, such as limited information, unclear participation channels, and academic pressure, persist [136]. Higher eHealth literacy may facilitate access to and comprehension of reliable health information, thereby enhancing the understanding of the significance of volunteer roles in public health efforts [137]. However, this association has been examined in only a few studies, indicating the need for further research to clarify the mechanisms and contextual factors involved.

### Relationship Between eHealth Literacy and Emotional Outcomes in Postsecondary Students

Our review found that higher eHealth literacy is positively associated with psychosocial wellness indicators, such as better mental health, resilience to peer pressure, and enhanced spiritual well-being. This relationship likely reflects individuals’ improved capacity to critically evaluate online health information, thereby reducing exposure to misinformation and related distress, which supports more informed health decisions and stronger psychosocial resilience [18,73]. Additionally, higher eHealth literacy appears linked to a stronger sense of coherence, as it enhances important sense of coherence components: comprehensibility (understanding health risks and information), manageability (confidence in addressing these risks), and meaningfulness (valuing engagement in health behaviors) [85]. These cognitive frameworks are vital for stress resilience and maintaining psychological balance.

For medical students, eHealth literacy may support the fulfillment of basic psychological needs outlined in the self-determination theory. Engaging with health information collaboratively fosters relatedness, while self-motivated use aligned with personal values satisfies autonomy. Additionally, acquiring and applying health information enhances competence, contributing to academic growth and professional identity development [77]. However, the relationship between eHealth literacy and overall well-being remains inconclusive. Some studies report positive associations, often linked to reduced COVID-19 fear and improved health information satisfaction, which may promote perceived control and self-care [65,74,76]. Yet, these findings are predominantly from the pandemic context, limiting generalizability. Moreover, discrepancies exist. One study found that only the personal relevance dimension of eHealth literacy was associated with well-being [79], while another observed no significant overall effect after adjusting for anxiety and sense of coherence [78]. Variations in measurement tools and analytic methods likely explain these inconsistent results, underscoring the need for further research using standardized assessments and robust analyses to clarify the impact of eHealth literacy on well-being.

Conversely, eHealth literacy has been shown to be negatively associated with adverse emotional outcomes, such as anxiety, cyberchondria, and distress related to online health information seeking, possibly because individuals with higher eHealth literacy are better able to access and use online mental health resources for emotion regulation and psychological adaptation [138].

The relationship between eHealth literacy and depression remains unclear. For example, the study by Tran et al [81] reported no significant association between increasing eHealth literacy scores and depression incidence, whereas the study by Xie et al [80] identified inadequate eHealth literacy as a significant risk factor for depression. Both used the same eHealth literacy tool, but differing depression measures and statistical approaches (treating eHealth literacy as continuous versus categorical) may explain these discrepancies. Thus, further research with standardized depression assessments and robust analytic methods is warranted to clarify this relationship.

Additionally, the link between eHealth literacy and fear of COVID-19 during the pandemic is inconclusive. This may be partly due to the widespread use of social media for public health functions such as information dissemination, real-time monitoring, and outbreak prediction [139], which have enhanced public knowledge throughout the pandemic [65]. Individuals with higher eHealth literacy tend to seek health information across diverse digital platforms and leverage social networks, potentially reducing fear [65]. However, external factors like rising case numbers and deaths may increase uncertainty and perceived threat, possibly offsetting eHealth literacy benefits. Further research is needed to better understand this complex relationship [140].

### Relationship Between eHealth Literacy and Behavioral Outcomes in Postsecondary Students

Our review found that higher eHealth literacy correlates with increased health-related social media use, both online and offline health information–seeking behaviors, information processing abilities, and effective use of mobile health apps among postsecondary students. These outcomes likely stem from students’ enhanced ability to locate, comprehend, and critically evaluate digital health information, which increases the perceived usefulness of digital tools and supports behavior change in line with the KAP model [128,134]. These students are thus more inclined to actively seek health information, process it efficiently, and use digital health tools effectively. Furthermore, eHealth literacy appears to be inversely related to mobile phone addiction, possibly due to stronger self-regulation and critical appraisal skills [91].

However, findings on the relationship between eHealth literacy and general social media use are mixed. While a study in Taiwan during the COVID-19 pandemic found no significant association [58], a prepandemic US study reported a positive correlation [87]. These inconsistencies may stem from differences in context, timing, and measurement methods. This suggests that the relationship is likely multifactorial and context-dependent. Future studies should adopt multidimensional assessments (considering frequency, intensity, motivation, content, and interaction patterns) across diverse populations and periods to clarify this association.

Regarding health care use, different dimensions of eHealth literacy show divergent associations. Luo et al [92] reported a negative association between functional eHealth literacy and the frequency of medical service use, possibly because individuals with stronger foundational skills can manage their health independently [141-143]. In contrast, interactive eHealth literacy was positively associated with the effective use of various health care providers and systems, as well as with more frequent service use. This may reflect the role of advanced cognitive and communication skills [144] in applying health information in personalized contexts and increasing decision-making confidence [145]. Moreover, greater information access may induce uncertainty or anxiety, leading to more frequent consultations with professionals [92]. Critical eHealth literacy has been linked to the use of diverse health care services and needs-based health care–seeking behaviors, as individuals with higher critical literacy are better at evaluating risks and benefits and advocating for their needs [92]. However, these findings are primarily drawn from a single study, and thus, further research with larger, more diverse samples is needed to validate these associations across different countries, academic disciplines, and educational levels.

In terms of healthy living, eHealth literacy is associated with better physical health, likely because individuals with higher application abilities are more capable of using online resources to create effective exercise plans, make informed decisions based on their health status, and identify credible information [93]. Consequently, students with higher eHealth literacy may have greater motivation and energy to adopt healthy behaviors [93]. While several studies have reported a positive relationship between eHealth literacy and self-care agency, most have focused on nursing or medical students. Only 1 study found a significant association in nursing students but not in nonhealth care students, possibly due to limited skills in searching, understanding, and evaluating online health information [94]. Further research is needed among nonhealth majors.

The relationship between eHealth literacy and healthy lifestyle behaviors is complex. Some studies report significant positive associations with specific behaviors, such as maintaining regular routines, practicing safe sex, and life appreciation. This may be because maintaining regular routines and safe sex are closely related to awareness of health risks and prevention, which are core competencies emphasized in eHealth literacy. Additionally, individuals with higher eHealth literacy are more likely to understand concepts related to positive psychology and life meaning, which can promote behaviors like life appreciation [51]. Many of these behaviors involve autonomous decision-making and can be adopted immediately upon accessing accurate information.

However, inconsistent findings have also been reported. For example, the study by Nam et al [67] found no significant correlation among Korean students and a negative association among Chinese students. Additionally, the relationship between eHealth literacy and other behaviors, such as sleep, nutrition, physical activity, substance avoidance, interpersonal relationships, health responsibility, and mental health, was mixed. This may be because these behaviors depend not only on an individual’s ability to obtain, understand, and apply health information (skills stronger among those with higher eHealth literacy) but also on external factors like resource availability and social context [109]. Therefore, examining eHealth literacy by its subdimensions helps clarify the mechanisms and boundary conditions that influence its role in promoting health behaviors, providing more targeted theoretical guidance for interventions.

Analyzing eHealth literacy by its subdimensions (functional, interactive, and critical) provides greater insights. Our findings indicate that critical eHealth literacy is more strongly associated with health-promoting behaviors than functional or interactive literacy. Critical literacy involves advanced cognitive skills, enabling individuals to evaluate information comprehensively, recognize risks and benefits, and advocate for themselves [146]. Therefore, students with high critical literacy are better equipped to engage in health-enhancing behaviors [20]. In contrast, functional and interactive literacy represent more basic skills that do not involve the same depth of processing [147]. It is not sufficient to merely access information, and critical evaluation and application are essential for informed decision-making. However, further research is needed to explore these relationships in diverse populations and contexts to better understand the specific mechanisms involved.

Regarding disease-related behaviors, eHealth literacy was positively associated with disease prevention and management behaviors, likely because individuals with higher literacy better locate, understand, and apply health information for informed decisions [122]. However, a negative association with HPV vaccination was observed, the reasons for which remain unclear. The study by Williams [114] involved diverse racial groups but did not analyze eHealth literacy subgroups and focused on university students likely beyond the recommended HPV vaccination age. Additionally, limited HPV knowledge and health care provider recommendations influenced vaccination uptake [114]. These factors suggest that the relationship between eHealth literacy and vaccination behavior is inconclusive, highlighting the need for further research across different populations and vaccine types.

During the pandemic, eHealth literacy was positively associated with several COVID-19 preventive behaviors, including handwashing, staying at home except for essential activities, quarantine participation, and vaccination, likely because individuals with higher literacy better identify and evaluate reliable information sources [81]. However, associations with physical distancing and mask wearing were inconsistent, and no links were found for avoiding crowded places or maintaining indoor ventilation. Jiang et al [117] reported no significant associations for these latter behaviors, possibly due to differences in country context, pandemic phase, or outbreak severity. Additionally, behaviors like mask wearing and distancing may be more influenced by cultural norms, public attitudes, and external regulations than by individual knowledge [148]. These findings indicate that the influence of eHealth literacy varies across behaviors and may be limited when actions are habitual or externally enforced. Further research should examine other factors interacting with eHealth literacy in public health emergencies.

eHealth literacy is positively associated with engagement in volunteer activities, possibly because individuals with higher literacy access and understand authoritative online information on public health, which may enhance their commitment to volunteering through the KAP pathway [134]. However, evidence is limited, and further research is needed to clarify this relationship. Similarly, eHealth literacy shows a positive correlation with clinical decision-making ability. This may be due to improved skills in using online resources and critically evaluating medical information, enabling more informed decisions [121]. Yet, this area remains underexplored and requires more investigation.

This study has several strengths. First, it applied the PHE model, which offers a comprehensive framework to explore the impact of eHealth literacy on the cognitive, emotional, and behavioral aspects of engagement. This allows for a deeper understanding of how eHealth literacy influences not only knowledge and behaviors but also motivation and psychological engagement among postsecondary students. Second, the inclusion of studies published in multiple languages broadens the evidence base, capturing diverse cultural and contextual factors that may affect eHealth literacy and its outcomes. This enhances the generalizability and applicability of the findings across different countries and populations.

However, this review is not without limitations. First, all included studies were cross-sectional in design, which limits the ability to infer causal relationships between eHealth literacy and health-related outcomes. To better understand the directionality and underlying mechanisms of these associations, future longitudinal and interventional studies are warranted. Second, although study selection and data extraction were conducted independently by 2 reviewers, the interrater reliability (eg, Cohen κ) was not formally recorded. While discrepancies were resolved through discussion and consensus, the lack of a quantified agreement metric may have limited the transparency and reproducibility of the review process. Future reviews should consider formally reporting interrater reliability to enhance methodological rigor. Third, a key limitation lies in the heterogeneity and limited replicability of the reported outcomes. Although over 100 health-related outcomes were identified, the majority were examined in only a single study, and most relied on self-reported rather than objective clinical measures. This diversity and methodological inconsistency hinder the comparability and synthesis of findings and may compromise the robustness and generalizability of the conclusions. To address this, future research should aim to adopt standardized outcome measures, include validated clinical indicators when feasible, and replicate studies across diverse populations to strengthen the cumulative evidence base in this field. Fourth, this review is limited by the variability in the measurement of eHealth literacy across the included studies. Different instruments, such as the eHEALS, EHLS, and DHLI, were applied, with each being based on distinct conceptual frameworks and comprising different item constructs. This heterogeneity in assessment tools may have introduced inconsistencies in the reported levels of eHealth literacy and their associations with health-related outcomes, complicating direct comparison and synthesis of the results. Future research should strive for consensus on standardized and comprehensive measurement approaches to improve comparability and advance the field.

### Conclusion

This systematic review comprehensively examined eHealth literacy levels among postsecondary students and assessed their associations with various cognitive, emotional, and behavioral outcomes. Overall, students’ eHealth literacy ranged from moderate-low to moderate-high levels. However, inconsistencies in measurement tools and scoring systems underscore the need for more standardized and validated assessment methods.

eHealth literacy demonstrated positive correlations with students’ health-related knowledge, self-efficacy, disease prevention behaviors, health attitudes, and attitudes toward electronic health information, highlighting its crucial role in promoting health cognition. Generally, eHealth literacy is positively associated with psychosocial well-being and positive emotions and negatively correlated with negative emotions. Nonetheless, its relationships with well-being, depression, and fear of COVID-19 remain inconclusive, as they are influenced by multiple external factors, warranting further in-depth investigation.

Moreover, while eHealth literacy generally correlates positively with the use of electronic information, its influence on health care service use and social media engagement appears more complex. Similarly, the relationship between eHealth literacy and healthy living is multifaceted. Although most studies report positive associations, healthy living behaviors are also shaped by other factors. Positive links were also observed between eHealth literacy and disease prevention practices, volunteerism, and clinical decision-making abilities.

In conclusion, enhancing eHealth literacy among university students is critical for improving their health management capabilities and overall quality of life. Future research should prioritize standardizing assessment criteria and further exploring the manifestations and mechanisms of eHealth literacy across diverse academic disciplines and cultural contexts, thereby informing more effective educational and health promotion strategies.

## Appendix

Multimedia Appendix 1 PRISMA (Preferred Reporting Items for Systematic Reviews and Meta-Analyses) checklist.

Multimedia Appendix 2 Detailed search strategy.

Multimedia Appendix 3 Overall characteristics of the 89 included studies.

Multimedia Appendix 4 Risk of bias assessment of the 89 included studies.

## Abbreviations

AXIS: Appraisal Tool for Cross-Sectional Studies
DHLI: Digital Health Literacy Instrument
eHEALS: e-Health Literacy Scale
EHLS: eHealth Literacy Scale
HPV: human papillomavirus
KAP: knowledge-attitude-practice
PHE: patient health engagement
