# Supplementary material for: eHealth Literacy and Its Outcomes Among Postsecondary Students: Systematic Review
Source: J Med Internet Res. 2025 Jul 2;27:e64489. doi: 10.2196/64489 (PMC12278882; doi:10.2196/64489)
Supplement: Multimedia Appendix 2 [file jmir_v27i1e64489_app2.docx]

**Multimedia** [**Appendix 2.**](https://www.ncbi.nlm.nih.gov/pmc/articles/PMC9926349/#app2) Detailed search strategy.

The search terms were about two domains (one was e-health literacy related, and the other was “relate” related), as below. Last search on 1^th^ July, 2024.

| #1 | Electronic health literacy  E-Health literacy  EHealth literacy  Technology health literacy  Digital health literacy  Mobile health literacy  MHealth literacy  Telehealth literacy  Telemedicine literacy  Internet health literacy  Internet based health literacy  Computer health literacy  Computer based health literacy  Web health literacy  Web based health literacy  Online health literacy  Online based health literacy | #2 | Relate  Relation  Relevant  Relevance  Associate  Association  Correlate  Correlation  Determinant  Outcome  Factor  Effect  Affect  Influence  Impact |
| --- | --- | --- | --- |

The search strategies and preliminary results in different databases were as follows:

1. **PubMed (n = 9240)**

| **Search** | **Details** | **Results** |
| --- | --- | --- |
| #1 | (("electronical"[All Fields] OR "electronically"[All Fields] OR "electronics"[MeSH Terms] OR "electronics"[All Fields] OR "electronic"[All Fields]) AND ("health literacy"[MeSH Terms] OR ("health"[All Fields] AND "literacy"[All Fields]) OR "health literacy"[All Fields])) OR ("e-Health"[All Fields] AND ("literacies"[All Fields] OR "literacy"[MeSH Terms] OR "literacy"[All Fields] OR "literacy s"[All Fields])) OR (("telemedicine"[MeSH Terms] OR "telemedicine"[All Fields] OR "ehealth"[All Fields]) AND ("literacies"[All Fields] OR "literacy"[MeSH Terms] OR "literacy"[All Fields] OR "literacy s"[All Fields])) OR (("biomedical technology"[MeSH Terms] OR ("biomedical"[All Fields] AND "technology"[All Fields]) OR "biomedical technology"[All Fields] OR ("technology"[All Fields] AND "health"[All Fields]) OR "technology health"[All Fields]) AND ("literacies"[All Fields] OR "literacy"[MeSH Terms] OR "literacy"[All Fields] OR "literacy s"[All Fields])) OR (("digital health"[MeSH Terms] OR ("digital"[All Fields] AND "health"[All Fields]) OR "digital health"[All Fields]) AND ("literacies"[All Fields] OR "literacy"[MeSH Terms] OR "literacy"[All Fields] OR "literacy s"[All Fields])) OR (("telemedicine"[MeSH Terms] OR "telemedicine"[All Fields] OR ("mobile"[All Fields] AND "health"[All Fields]) OR "mobile health"[All Fields]) AND ("literacies"[All Fields] OR "literacy"[MeSH Terms] OR "literacy"[All Fields] OR "literacy s"[All Fields])) OR (("mhealth s"[All Fields] OR "telemedicine"[MeSH Terms] OR "telemedicine"[All Fields] OR "mhealth"[All Fields]) AND ("literacies"[All Fields] OR "literacy"[MeSH Terms] OR "literacy"[All Fields] OR "literacy s"[All Fields])) OR (("telehealth s"[All Fields] OR "telemedicine"[MeSH Terms] OR "telemedicine"[All Fields] OR "telehealth"[All Fields]) AND ("literacies"[All Fields] OR "literacy"[MeSH Terms] OR "literacy"[All Fields] OR "literacy s"[All Fields])) OR (("telemedicine"[MeSH Terms] OR "telemedicine"[All Fields] OR "telemedicine s"[All Fields]) AND ("literacies"[All Fields] OR "literacy"[MeSH Terms] OR "literacy"[All Fields] OR "literacy s"[All Fields])) OR (("internet"[MeSH Terms] OR "internet"[All Fields] OR "internet s"[All Fields] OR "internets"[All Fields]) AND ("health literacy"[MeSH Terms] OR ("health"[All Fields] AND "literacy"[All Fields]) OR "health literacy"[All Fields])) OR (("internet"[MeSH Terms] OR "internet"[All Fields] OR "internet s"[All Fields] OR "internets"[All Fields]) AND ("based"[All Fields] OR "basing"[All Fields]) AND ("health literacy"[MeSH Terms] OR ("health"[All Fields] AND "literacy"[All Fields]) OR "health literacy"[All Fields])) OR (("computability"[All Fields] OR "computable"[All Fields] OR "computating"[All Fields] OR "computation"[All Fields] OR "computational"[All Fields] OR "computations"[All Fields] OR "compute"[All Fields] OR "computed"[All Fields] OR "computer s"[All Fields] OR "computers"[MeSH Terms] OR "computers"[All Fields] OR "computer"[All Fields] OR "computes"[All Fields] OR "computing"[All Fields] OR "computional"[All Fields]) AND ("health literacy"[MeSH Terms] OR ("health"[All Fields] AND "literacy"[All Fields]) OR "health literacy"[All Fields])) OR (("computability"[All Fields] OR "computable"[All Fields] OR "computating"[All Fields] OR "computation"[All Fields] OR "computational"[All Fields] OR "computations"[All Fields] OR "compute"[All Fields] OR "computed"[All Fields] OR "computer s"[All Fields] OR "computers"[MeSH Terms] OR "computers"[All Fields] OR "computer"[All Fields] OR "computes"[All Fields] OR "computing"[All Fields] OR "computional"[All Fields]) AND ("based"[All Fields] OR "basing"[All Fields]) AND ("health literacy"[MeSH Terms] OR ("health"[All Fields] AND "literacy"[All Fields]) OR "health literacy"[All Fields])) OR ("web"[All Fields] AND ("health literacy"[MeSH Terms] OR ("health"[All Fields] AND "literacy"[All Fields]) OR "health literacy"[All Fields])) OR ("web"[All Fields] AND ("based"[All Fields] OR "basing"[All Fields]) AND ("health literacy"[MeSH Terms] OR ("health"[All Fields] AND "literacy"[All Fields]) OR "health literacy"[All Fields])) OR ("online"[All Fields] AND ("health literacy"[MeSH Terms] OR ("health"[All Fields] AND "literacy"[All Fields]) OR "health literacy"[All Fields])) OR ("online"[All Fields] AND ("based"[All Fields] OR "basing"[All Fields]) AND ("health literacy"[MeSH Terms] OR ("health"[All Fields] AND "literacy"[All Fields]) OR "health literacy"[All Fields])) | 13,576 |
| #2 | "relate"[All Fields] OR ("family"[MeSH Terms] OR "family"[All Fields] OR "relation"[All Fields] OR "relatability"[All Fields] OR "relatable"[All Fields] OR "related"[All Fields] OR "relates"[All Fields] OR "relating"[All Fields] OR "relational"[All Fields] OR "relations"[All Fields]) OR ("relevance"[All Fields] OR "relevances"[All Fields] OR "relevancies"[All Fields] OR "relevancy"[All Fields] OR "relevant"[All Fields]) OR ("relevance"[All Fields] OR "relevances"[All Fields] OR "relevancies"[All Fields] OR "relevancy"[All Fields] OR "relevant"[All Fields]) OR ("associate"[All Fields] OR "associated"[All Fields] OR "associates"[All Fields] OR "associating"[All Fields] OR "association"[MeSH Terms] OR "association"[All Fields] OR "associations"[All Fields]) OR ("associate"[All Fields] OR "associated"[All Fields] OR "associates"[All Fields] OR "associating"[All Fields] OR "association"[MeSH Terms] OR "association"[All Fields] OR "associations"[All Fields]) OR ("correlate"[All Fields] OR "correlated"[All Fields] OR "correlates"[All Fields] OR "correlating"[All Fields] OR "correlation"[All Fields] OR "correlation s"[All Fields] OR "correlations"[All Fields] OR "correlative"[All Fields] OR "correlatives"[All Fields]) OR ("correlate"[All Fields] OR "correlated"[All Fields] OR "correlates"[All Fields] OR "correlating"[All Fields] OR "correlation"[All Fields] OR "correlation s"[All Fields] OR "correlations"[All Fields] OR "correlative"[All Fields] OR "correlatives"[All Fields]) OR ("analysis"[MeSH Subheading] OR "analysis"[All Fields] OR "determination"[All Fields] OR "determinant"[All Fields] OR "determinants"[All Fields] OR "determinate"[All Fields] OR "determinated"[All Fields] OR "determinates"[All Fields] OR "determinating"[All Fields] OR "determinations"[All Fields] OR "determine"[All Fields] OR "determined"[All Fields] OR "determines"[All Fields] OR "determining"[All Fields]) OR ("outcome"[All Fields] OR "outcomes"[All Fields]) OR ("factor"[All Fields] OR "factor s"[All Fields] OR "factors"[All Fields]) OR ("effect"[All Fields] OR "effecting"[All Fields] OR "effective"[All Fields] OR "effectively"[All Fields] OR "effectiveness"[All Fields] OR "effectivenesses"[All Fields] OR "effectives"[All Fields] OR "effectivities"[All Fields] OR "effectivity"[All Fields] OR "effects"[All Fields]) OR ("affect"[MeSH Terms] OR "affect"[All Fields] OR "affects"[All Fields] OR "affected"[All Fields] OR "affecteds"[All Fields] OR "affecting"[All Fields]) OR ("influence"[All Fields] OR "influenced"[All Fields] OR "influences"[All Fields] OR "influencing"[All Fields]) OR ("impact"[All Fields] OR "impactful"[All Fields] OR "impacting"[All Fields] OR "impacts"[All Fields] OR "tooth, impacted"[MeSH Terms] OR ("tooth"[All Fields] AND "impacted"[All Fields]) OR "impacted tooth"[All Fields] OR "impacted"[All Fields]) | 25,845,614 |
| #3 | #1 AND #2 | 12,302 |
| #4 | #1 AND #2 AND species(Humans) | 9,629 |
| #5 | #1 AND #2 AND species(Humans), Time span=2006-2024 | 9,240 |

1. **Web of Science (n = 13616)**

| **Search** | **Details** | **Results** |
| --- | --- | --- |
| #1 | **((((((((((((((((TS=(electronic health literacy)) OR TS=(e-Health literacy)) OR TS=(eHealth literacy)) OR TS=(technology health literacy)) OR TS=(digital health literacy)) OR TS=(mobile health literacy)) OR TS=(mHealth literacy)) OR TS=(telehealth literacy)) OR TS=(telemedicine literacy)) OR TS=(internet health literacy)) OR TS=(internet based health literacy)) OR TS=(computer health literacy)) OR TS=(computer based health literacy)) OR TS=(web health literacy)) OR TS=(web based health literacy)) OR TS=(online health literacy)) OR TS=(online based health literacy)** and **Preprint Citation Index** (Exclude – Database) | 21,559 |
| #2 | ((((((((((((((AB=(relate)) OR AB=(relation)) OR AB=(relevant)) OR AB=(relevance)) OR AB=(associate)) OR AB=(association)) OR AB=(correlate)) OR AB=(correlation)) OR AB=(determinant)) OR AB=(outcome)) OR AB=(factor)) OR AB=(effect)) OR AB=(affect)) OR AB=(influence)) OR AB=(impact) | 34,111,287 |
| #3 | #1 and #2 | 16,233 |
| #4 | #1 and #2 and PY=(2006-2024) | 15,356 |
| #5 | #1 AND #2 AND #3 and PY=(2006-2024)   and Letter or Review Article or Editorial Material or Retracted Publication or Item Withdrawal or Reference Material (Exclude – Document Types) | 13,616 |

1. **CINAHL Complete, accessed via EBSCOhost platform (n = 2449)**

| **Search** | **Details** | **Restrictive conditions** | **Search method** | **Results** |
| --- | --- | --- | --- | --- |
| S1 | TX electronic health literacy OR TX e-Health literacy OR TX eHealth literacy OR TX technology health literacy OR TX digital health literacy OR TX mobile health literacy OR TX mHealth literacy OR TX telehealth literacy OR TX telemedicine literacy OR TX internet health literacy OR TX internet based health literacy OR TX computer health literacy | Expanders - Also search within the full text of the articles; Apply equivalent subjects  Search modes-Boolean/Phrase | Search Screen - Advanced Search | 2,384 |
| S2 | TX computer based health literacy OR TX web health literacy OR TX web based health literacy OR TX online health literacy OR TX online based health literacy | Expanders - Also search within the full text of the articles; Apply equivalent subjects  Search modes-Boolean/Phrase | Search Screen - Advanced Search | 658 |
| S3 | S1 OR S2 | Expanders - Also search within the full text of the articles; Apply equivalent subjects  Search modes-Boolean/Phrase | Search Screen - Advanced Search | 2,749 |
| S4 | TX relate OR TX relation OR TX relevant OR TX relevance OR TX associate OR TX association OR TX correlate OR TX correlation OR TX determinant OR TX outcome OR TX factor OR TX effect | Expanders - Also search within the full text of the articles; Apply equivalent subjects  Search modes-Boolean/Phrase | Search Screen - Advanced Search | 5,183,523 |
| S5 | TX affect OR TX influence OR TX impact | Expanders - Also search within the full text of the articles; Apply equivalent subjects  Search modes-Boolean/Phrase | Search Screen - Advanced Search | 1,599,139 |
| S6 | S4 OR S5 | Expanders - Also search within the full text of the articles; Apply equivalent subjects  Search modes-Boolean/Phrase | Search Screen - Advanced Search | 5,377,666 |
| S7 | S3 AND S6 | Expanders - Also search within the full text of the articles; Apply equivalent subjects  Search modes-Boolean/Phrase | Search Screen - Advanced Search | 2,497 |
| S8 | S3 AND S6 | Limiters - Publication Date: 20060101-20241231  Expanders - Also search within the full text of the articles; Apply equivalent subjects  Search modes - Boolean/Phrase | Search Screen - Advanced Search | 2,449 |

1. **Embase (n = 662)**

| **Search** | **Details** | **Results** |
| --- | --- | --- |
| #1 | 'electronic health literacy' OR 'e-health literacy'/exp OR 'e-health literacy' OR 'ehealth literacy'/exp OR 'ehealth literacy' OR 'technology health literacy' OR 'digital health literacy' OR 'digit health literacy' OR 'mobile health literacy' OR 'mhealth literacy' OR 'm-health literacy' OR 'telehealth literacy'/exp OR 'telehealth literacy' OR 'telemedicine literacy' OR 'internet health literacy' OR 'internet-based health literacy' OR 'internet based health literacy' OR 'computer health literacy' OR 'computer based health literacy' OR 'computer-based health literacy' OR 'web health literacy' OR 'web based health literacy' OR 'web-based health literacy' OR 'online health literacy'/exp OR 'online health literacy' OR 'online based health literacy' OR 'online-based health literacy' | 1,334 |
| #2 | ((((((((((((((('relate') OR ('relation')) OR ('relevant')) OR ('relevance')) OR ('associate')) OR ('association')) OR ('correlate')) OR ('correlation')) OR ('determinant')) OR ('outcome')) OR ('factor')) OR ('effect')) OR ('affect')) OR ('influence')) OR ('impact')) | 20,869,967 |
| #3 | #1 AND #2 | 887 |
| #4 | #1 AND #2 AND ([article]/lim OR [article in press]/lim OR [conference paper]/lim OR [data papers]/lim OR [preprint]/lim) | 672 |
| #5 | #1 AND #2 AND ([article]/lim OR [article in press]/lim OR [conference paper]/lim OR [data papers]/lim OR [preprint]/lim) AND [humans]/lim | 662 |
| #6 | #1 AND #2 AND ([article]/lim OR [article in press]/lim OR [conference paper]/lim OR [data papers]/lim OR [preprint]/lim) AND [humans]/lim AND [2006-2024]/py | 662 |

1. **Cochrane library (n = 1881)**

| **Search** | **Details** | **Results** |
| --- | --- | --- |
| #1 | (electronic health literacy) OR (e-Health literacy) OR (eHealth literacy) OR (technology health literacy) OR (digital health literacy) (Word variations have been searched) | 1204 |
| #2 | (mobile health literacy) OR (mHealth literacy) OR (telehealth literacy) OR (telemedicine literacy) OR (internet health literacy) (Word variations have been searched) | 1,139 |
| #3 | (internet based health literacy) OR (computer health literacy) OR (computer based health literacy) OR (web health literacy) OR (web based health literacy) (Word variations have been searched) | 1,085 |
| #4 | (online health literacy) OR (online based health literacy) (Word variations have been searched) | 714 |
| #5 | #1 OR #2 OR #3 OR #4 | 2,141 |
| #6 | (relate) OR (relation) OR (relevant) OR (relevance) OR (associate) (Word variations have been searched) | 2,161,365 |
| #7 | (association) OR (correlate) OR (correlation) OR (determinant) OR (outcome) (Word variations have been searched) | 1,230,763 |
| #8 | (factor) OR (effect) OR (affect) OR (influence) OR (impact) (Word variations have been searched) | 1,472,646 |
| #9 | #6 OR #7 OR #8 | 2,161,365 |
| #10 | #5 AND #9 | 2,141 |
| #11 | #5 AND #9 with Publication Year from 2006 to 2024, with Cochrane Library publication date Between Jan 2006 and Dec 2024, in Trials | 1,881 |

1. **APA PsycInfo and APA PsycArticles(n=13534)**

| **Search** | **Details** | **Restrictive conditions** | **Search method** | **Results** |
| --- | --- | --- | --- | --- |
| S1 | TX electronic health literacy OR TX e-Health literacy OR TX eHealth literacy OR TX technology health literacy OR TX digital health literacy OR TX mobile health literacy OR TX mHealth literacy OR TX telehealth literacy OR TX telemedicine literacy OR TX internet health literacy OR TX internet based health literacy OR TX computer health literacy | Expanders - Apply equivalent subjects  Search modes - Find all my search terms | Search Screen - Advanced Search  Database - APA PsycInfo | 17,167 |
| S2 | TX computer based health literacy OR TX web health literacy OR TX web based health literacy OR TX online health literacy OR TX online based health literacy | Expanders - Apply equivalent subjects  Search modes - Find all my search terms | Search Screen - Advanced Search  Database - APA PsycInfo | 5,676 |
| S3 | S1 OR S2 | Expanders - Apply equivalent subjects  Search modes - Find all my search terms | Search Screen - Advanced Search  Database - APA PsycInfo | 17,172 |
| S4 | TX relate OR TX relation OR TX relevant OR TX relevance OR TX associate OR TX association OR TX correlate OR TX correlation OR TX determinant OR TX outcome OR TX factor OR TX effect | Expanders - Apply equivalent subjects  Search modes - Find all my search terms | Search Screen - Advanced Search  Database - APA PsycInfo | 3,698,504 |
| S5 | TX affect OR TX influence OR TX impact | Expanders - Apply equivalent subjects  Search modes - Find all my search terms | Search Screen - Advanced Search  Database - APA PsycInfo | 1,303,529 |
| S6 | S4 OR S5 | Expanders - Apply equivalent subjects  Search modes - Find all my search terms | Search Screen - Advanced Search  Database - APA PsycInfo | 4,010,526 |
| S7 | S3 AND S6 | Expanders - Apply equivalent subjects  Search modes - Find all my search terms | Search Screen - Advanced Search  Database - APA PsycInfo | 14,385 |
| S8 | S3 AND S6 | Limiters - Publication Year: 2006-2024  Expanders - Apply equivalent subjects  Search modes - Find all my search terms | Search Screen - Advanced Search  Database - APA PsycInfo | 13,534 |

1. **China National Knowledge Infrastructure(CNKI)(n=143)**

| **Search** | **Details** | **Results** |
| --- | --- | --- |
| #1 | (Full text: Electronic Health Literacy) OR (Full text: Digital Health Literacy)  Source category: Peking University Core | 119 |
| #2 | (Full text: Electronic Health Literacy) OR (Full text: Digital Health Literacy)  Source category: dissertation | 24 |

1. **Wanfang(n=223)**

| **Search** | **Details** | **Results** |
| --- | --- | --- |
| #1 | (Theme: Electronic Health Literacy) OR (Theme: Digital Health Literacy)  Source category: Peking University Core | 101 |
| #2 | (Theme: Electronic Health Literacy) OR (Theme: Digital Health Literacy)  Source category: dissertation | 122 |

1. **Base(n=3413)**

("electronic health literacy" OR "e-Health literacy" OR "eHealth literacy" OR "technology health literacy" OR "digital health literacy" OR "mobile health literacy" OR "mHealth literacy" OR "telehealth literacy" OR "telemedicine literacy" OR "internet health literacy" OR "internet based health literacy" OR "computer health literacy" OR "computer based health literacy" OR "web health literacy" OR "web based health literacy" OR "online health literacy" OR "online based health literacy") AND ("relate" OR "relation" OR "relevant" OR "relevance" OR "associate" OR "association" OR "correlate" OR "correlation" OR "determinant" OR "outcome" OR "factor" OR "effect" OR "affect" OR "influence" OR "impact" ) 2006-2024

**10. Opengrey(n=5)**

| **Search strategy(s) including how items were selected.**  *Search strategies:* | **Results** |
| --- | --- |
| electronic health literacy | **0** |
| e-Health literacy | 2 |
| eHealth literacy | 2 |
| technology health literacy | 0 |
| digital health literacy | 1 |
| mobile health literacy | 0 |
| mHealth literacy | 0 |
| telehealth literacy | 0 |
| telemedicine literacy | 0 |
| internet health literacy | 0 |
| internet based health literacy | 0 |
| computer health literacy | 0 |
| computer based health literacy | 0 |
| web health literacy | 0 |
| web based health literacy | 0 |
| online health literacy | 0 |
| online based health literacy | 0 |
