# Supplementary material for: eHealth Literacy and Its Outcomes Among Postsecondary Students: Systematic Review
Source: J Med Internet Res. 2025 Jul 2;27:e64489. doi: 10.2196/64489 (PMC12278882; doi:10.2196/64489)
Supplement: Multimedia Appendix 3 [file jmir_v27i1e64489_app3.doc]

**Multimedia Appendix 3.** Overall characteristics of the 89 included studies.

| **Citation in this file** | **Citation corresponding to the manuscript reference** | **Author(year of publication)** | **Characteristics of participants** | | | | | **E-Health literacy measuring instrument** | **E-Health literacy level** | **Outcomes** | **Outcome variables measurements** |
| --- | --- | --- | --- | --- | --- | --- | --- | --- | --- | --- | --- |
|  |  |  | **Country** | **Sample size** | **Population type** | **Age** | **female n (%)** |  |  |  |  |
| [1] | [19] | Yuan T, et al. (2020) | China | 613 | Undergraduate students | 20.88 ± 55.00 ( 17.00~25.00) | 418  (68.2) | eHEALS | 30.45±6.90 | (1)COVID-19 Knowledge  (2)COVID-19 specific precautionary behaviors (3)Attitude to prevention and control of COVID-19 | Self-designed items based on previous literature |
| [2] | [20] | Yang SC et al. (2017) | China(Taiwan) | 556 | College Students | / | 450  (80.9) | EHLS | / | (1)Healthy lifestyle Behavior  (2)Physical activity  (3)Nutrition (4)Health responsibility behaviors for maintaining personal and public hygiene (5)Interpersonal relationships (6)Stress management (7)Life satisfaction and positive growth behaviors | Health-promoting Lifestyle Scale |
| [3] | [21] | Patil U et al. (2021) | America | 256 | College students | 23.90 ± 4.30 | 108  (42.0) | Modified DHLI | 2.99 ± 0.51 | (1)Attitudes towards the handling of the COVID-19 (2)COVID-19 vaccine intention (3)COVID-19 specific precautionary behaviors  (4)COVID-19 would likely be contracted (5)COVID-19 would severely impact their life | Adaptedt from COVID-HL Consortium |
| [4] | [26] | Tsukahara S et al. (2020) | Japan | 3183 | Undergraduate and Graduate students | / | 878  (27.6) | eHEALS | 23.6±6.8 | (1)Physical activity (2)Regular breakfast habit (3)Smoking (4)Alcohol  (5)Sufficient sleep | Self-designed items based on previous literature |
| [5] | [28] | Lotto M et al. (2023) | Brazil | 521 | Undergraduate students | 21.20 ±3.70 | 318  (62.0) | eHEALS | 28.0±5.53 | Health information seeking behavior | Self-designed items based on previous literature |
| [6] | [29] | [Ana Luiza Dallora et al. (2024](https://pubmed.ncbi.nlm.nih.gov/?term=Dallora AL[Author])) | Swedish+Polish | 646 | Undergraduate nursing students | 23.9± 6.39 | 555  (85.9) | eHEALS | 3.95±0.75 | (1)Technology enthusiasm (2)Technology anxiety | the Technophilia instrument (TechPH) |
| [7] | [38] | Oducado et al. (2020) | Filipino | 66 | Undergraduate nursing students | 20.05± 1.09 | 46  (69.7) | eHEALS | 4.02±0.71 | (1)Seeking health information online (2)Perceived usefulness of the Internet (3)Perceived importance of accessing health resources online | Self-designed items based on previous literature |
| [8] | [39] | Qin N et al. (2021) | China | 5,641 | Undergraduate students Graduate students PhD students | Male：25.95±4.07  Female:26.21±3.83 | 2,312 (40.99) | eHEALS | 30.68±7.16 | Protective behaviors following vaccination | Self-designed items based on previous literature |
| [9] | [40] | Li S et al. (2021) | China | 1873 | Undergraduate students | 19.6±1.8(18~25) | 904  (48.3) | eHEALS | / | (1)COVID-19 knowledge  (2)COVID‐19 related preventive behaviors (3)COVID-19 specific precautionary behaviors | Self-designed items based on previous literature |
| [10] | [41] | Qin N et al. (2021) | China | 3785 | Undergraduate students Graduate students PhD students | 20.90±3.14 | 1524  (40.26) | the mobile eHealth Literacy Scale (m-eHEALS) | 46.61±8.16 | (1)COVID-19 vaccination knowledge (2)Attitudes towards the necessity and benefits of COVID-19 vaccination (3)COVID-19 vaccination information seeking, sharing, vaccination, and post-vaccination behavior | Self-designed items based on previous literature |
| [11] | [42] | Mai, J. R et al. （2022） | China | 1 409 | Undergraduate nursing students | 20.70±1.40 （17.00~25.00） | 1 204（85.5) | eHEALS | 29.6±5.0 | Infectious disease health literacy | Self-designed items based on previous literature |
| [12] | [43] | Aslantekin-Özcoban F et al. (2021) | Turkey | 1003 | University students in their senior year | 21.42 ±2.39 | 716  (74.6) | eHEALS | 25.68 ± 7.0 | Emergency contraception knowledge | Emergency contraception (EC) Knowledge Test |
| [13] | [44] | Kılınç İşleyen E et al. (2024) | Turkey | 589 | University students | 21.57±1.64  (18.00～24.00) | 589  (100.0) | eHEALS | 28.44 ±5.80 | (1)Knowledge about cervical cancer and human papillomavirus (2)Perceived sensitivity and perceived seriousness of human papillomavirus and cervical cancer | Cervical cancer and human papillomavirus awareness questionnaire |
| [14] | [45] | Zhang S et al. (2024) | China | 385 | Undergraduate nursing students | 21.28 ± 1.23 (18.00～26.00) | 354  (91.9) | eHEALS | 30.59 ± 6.12 | (1)Health self-management competence (2)Mental health literacy | (1)The Adults Health Self-Management Skill Rating Scale (AHSMSRS) (2)Adolescent mental health literacy assessment scale |
| [15] | [46] | Mayukh NJJoC (2024) | Malaysia | 168 | University Students | 18.00 ± 33.00 | 125  （74.4） | eHEALS | 3.60±0.86 | (1)Self-efficacy (2)Cyberchondriac  (3)Health information-seeking behaviour | (1)General Self-Efficacy Scale (GSE) (2)the shortened Cyberchondria Severity Scale (CSS-12) (3)Self-designed items based on previous literature |
| [16] | [47] | Bao, X. L et al. (2022) | China | 971 | Associate degree students Undergraduate students Graduate students PhD students | — | 412  （42.4） | eHEALS | / | (1)Positive attitude to prevention and control of COVID-19 (2)Generalized self-efficacy (3)Changes to a healthy lifestyle | (1)COVID-19 Prevention and Control Cognition Questionnaire (2)General Self-Efficacy Scale（GSES) (3)Healthy Lifestyle Scale for University Students |
| [17] | [48] | Sögüt S et al. (2022) | Turkey | 578 | Midwifery Students | 21.00±1.83 | 578  (100.0) | eHEALS | 28.44±7.13 | Online Technologies Self-Efficacy | Online Technologies Self-Efficacy Scale |
| [18] | [49] | Sun H(2022) | China | 958 | Undergraduate nursing students | — | 723  (75.5) | Electronic media health literacy scale | 74.01 ± 13.36 | (1)Confidence in their social media ability (2)Willing to engage in health communication | (1)Social Media Self-efficacy Scale (2)The Health Communication Intention Questionnaire |
| [19] | [50] | Turan N et al. (2021) | Turkey | 205 | Undergraduate nursing students | 20.66±1.92 | 185  （90.2） | eHEALS | 29.50±5.02 | (1)Healthy Lifestyle Behavior (2)Life satisfaction and positive growth behaviors (3)Interpersonal relations (4)Stress management (5)Spiritual health (6)Health responsibility behaviors for maintaining personal and public hygiene (7)Physical activities  (8)Nutrition | the Adolescent Lifestyle Profile Scale |
| [20] | [51] | Pisl V et al. (2021) | Czechia | 866 | University students of medicine, pedagogy and law | Mean age: 23.58 | 621  (71.71) | eHEALS | 3.85±0.82 | (1)COVID-19 is a hoax (2)COVID-19 was created | (1)Hoax scale (HOAX)  (2)The human-made scale (CREATED) |
| [21] | [52] | Zadeh Kh et al. (2023) | Iran | 362 | Graduate students PhD students | 25.00～35.00 | 221  (61.05) | eHEALS | / | (1)Future Anxiety (2)COVID-19-related Conspiracy Belief | (1)Adapted from Future Anxiety scale (2)A COVID-19-related Conspiracy Belief Scale (CCBS) |
| [22] | [53] | Chen, Y. N et al. （2023） | China | 419 | Undergraduate students graduate students | 18.00～26.00 | 419  (100.0) | Modified eHEALS | 2.38 ±0.75 | (1)Perceived sensitivity and perceived seriousness of cervical cancer  (2)Seeking and obtaining information about cervical cancer | Self-designed items based on previous literature |
| [23] | [54] | Chun, H. R et al. (2021) | South Korea | 604 | Undergraduate students | / | 458  （75.8） | DHLI | 2.98±0.42 | (1)Participation in COVID-19 quarantine measures (2)COVID-19 vaccination intention | Self-designed items based on previous literature |
| [24] | [55] | Pisl V et al. (2021) | Czechia | 866 | University students of medicine, pedagogy and law | mean age 23.58 | 621  (71.71) | eHEALS | 3.85±0.82 | COVID-19 vaccination intention | Self-designed items based on previous literature |
| [25] | [56] | Kıbrıs Ş et al. (2023) | Turkey | 28,80 | University students | ≦26.00 | 230  （33.0） | eHEALS | 28.80±4.945 | Health perception | The Perception of Health Scale (PHS) |
| [26] | [57] | Liao LL et al. (2024) | China | 1571 | Undergraduate students | 20.75 ± 1.39 (18～27） | 1000  (63.65) | eHEALS | / | (1)E-cigarettes risk perception (2)E-cigarettes benefit perception (3)Social media usage | (1)(2)Adaptedt from The E-cigarette Risk and Benefit Perception Scale (3)Self-designed items based on previous literature |
| [27] | [58] | Fehér A et al. (2021) | Hungary | 612 | Vocational students Undergraduate students Graduate students | ≥18.00 | 372  (60.8) | EHLS | / | Attitudes toward healthy nutrition | Self-designed items based on previous literature |
| [28] | [59] | Noh M Y (2021) | South Korea | 272 | Associate degree students | 19.41±1.17 | 272  (100.0) | eHEALS | First year: 3.35±0.72 Second year: 3.63±0.64 | Attitudes towards exercise | Exercise Self-Schemata |
| [29] | [60] | Britt RK et al. (2017) | America | 420 | Midwestern university | 20.48± 2.14 (18.00～35.00) | / | eHEALS | 3.99±0 .71 | (1)Healthy Lifestyle Behavior (2)Online healthy lifestyle information seeking behavior  (3)Future health maintenance attitudes (4)Attitudes toward seeking and utilizing online health information in the future | Self-designed items based on the American College Health Association (ACHA) |
| [30] | [61] | Yan X D et al. （2018） | China | 398 | Associate degree student or Vocational student Undergraduate students Graduate students and above | ≦49.00 | 219  (55.03) | Modified eHEALS | Healthcare participants: 4.94±0.97 Non-healthcare participants: 4.19±0.88 | (1)Trust in mobile health software (2)Satisfaction with mobile health software | Self-designed items based on previous literature |
| [31] | [62] | Wang X et al. (2022) | China | 289 | College students | 22.46 ±1.94  (18.00～26.00 ) | 163  (56.40) | eHEALS | 26.92±5.82 | (1)Perceived usefulness of the internet  (2)Health lifestyle Behavior  (3)Online health information seeking behavior (4)Health information seeking inclination | (1)(3)(4)Self-designed items based on previous literature (2)Health-Promoting Lifestyle Profile (HPLP-II) |
| [32] | [63] | Chen SC et al. （2022） | China Taiwan | 1631 | Undergraduate students Graduate students PhD students | 22.60 ± 4.70 | 1193  (73.1) | DHLI | 3.1 ± 0.4 | (1)Future anxiety (2)Fear of COVID-19 (3)Satisfaction with online COVID-19 information | (1)Five-item future dark scale (2)the fear of COVID-19（F-CoV ）scale  (3)Self-designed items based on previous literature |
| [33] | [64] | Chen SC et al. (2023) | China Taiwan | 1631 | Undergraduate students Graduate students PhD students | 22.60 ± 4.70 | 1193 （73.1） | Modified DHLI | 3.1 ± 0.4 | （1）Well being （2）Fear of COVID-19 （3）Satisfaction with online COVID-19 information （4）Perceived importance of accessing health resources online | (1）WHO-5 well-being index （2）the fear of COVID-19（F-CoV ）scale (3)(4)Self-designed items based on previous literature |
| [34] | [65] | Kim J O (2017) | South Korea | 321 | Undergraduate students majoring in Health Information Management | / | 237（73.8） | eHEALS | Male: 3.75±0.63 Female: 3.42±0.51 | (1)Trust in online health information  (2)Attitudes towards Internet medical advertisement | (1)Scale develped by Lim Woo-ryeong (2)Attitudes Toward Medical Advertising scale developed by Han Su-yeon |
| [35] | [66] | Nam Y H et al. (2020) | South Korea | 240 | Undergraduate students | / | 182  (75.8) | Modified EHLS | Average: 3.27±.64 Korean student: 3.57±0.64 Chinese student：2.98±0.51 | (1)Trust in online health information   (2)Healthy Lifestyle Behavior (3)Accessing and using electronic health information ability | (1)Adapted from the existing literature  (2)Health Behavior Scale(HBS) (3)Self-designed items based on previous literature |
| [36] | [67] | Masilamani V et al. （2020） | India | 427 | College students | 19.52±1.44 (18.00～21.00) | 208  (48.6) | Modified eHEALS | 3.998±0.507 | Positive attitudes toward seeking and utilizing online health information | Adapted items based on previous literature |
| [37] | [68] | Kim H S et al. (2021) | South Korea | 152 | Undergraduate students | / | 117  (77.0) | Lee Sang-rok's e-Health Literacy Scale (2018) | 3.45±0.56 | (1)Need for volunteering (2)Volunteer Activity Status (3)Volunteer Activation | Self-designed items based on previous literature |
| [38] | [69] | Kuang H D et al. （2023） | China | 2239 | Associate degree student or Vocational student Undergraduate students Graduate students | 20.01±1.59 | 1223  (54.6) | eHEALS | 27.49±6.23 | (1)Online psychological help-seeking behavior (2)Mental health | (1)Self-designed items based on previous literature (2)Simple Mental Status Assessment Scale |
| [39] | [70] | Amoah PA et al. （2021） | China (Hong Kong and Macao) | 801 | University students | ≥16.00 | / | Modified DHLI | Average score: 2.86 | Mental health | the World Health Organization-5 Well-being Index |
| [40] | [71] | Chen W et al. (2020) | China | 491 | Associate degree student Undergraduate students Graduate students PhD students  above | / | 256  (52.14) | eHEALS | / | (1)Online mental health information seeking behavior (2)Mental health | (1)Adapted from the existing literature (2)Adapted from the hospital anxiety and depression scale |
| [41] | [72] | Xu G et al. (2022) | China | 515 | Undergraduate students | 19.27± 1.21 (18.00～21.00） | 393  (76.3) | The eHealth Literacy Scale for College Students | 62.97 ± 13.78 | (1)Health Self-Management (2)Resistance to Peer Influence | (1)Adults’ Health Self-Management Ability Assessment Scale (2)The Chinese Version of Resistance to Peer Influence Scale (RPIS) |
| [42] | [73] | Rivadeneira MF et al. (2022) | Ecuador | 917 | Undergraduate  Graduate students Other (a.e. PhD students) | / | 557  (60.74) | Digital Health Literacy with respect to COVID-19 | 2.9±0.5 | Wellbeing | World Health Organization (WHO) Wellbeing Scale |
| [43] | [74] | Choi S (2024) | America | 702 | College students | 24.33±3.64 (18.00～35.00) | 360  (51.3) | eHEALS | 28.65 ±6.02 | Well being | Flourishing Scale (FS) |
| [44] | [75] | Rivadeneira MF et al. (2022) | Ecuador | 917 | Undergraduate  Graduate students Other (a.e. PhD students) | / | 557  (60.74) | Digital Health Literacy with respect to COVID-19 | 2.9±0.5 | (1)Subjective social perception (2)Well-being | (1)MacArthur methodology  (2)the World Health Organization (WHO) well-being scale |
| [45] | [76] | Ha L N et al. (2023) | China | 2206 | Undergraduate medicine students Graduate medicine students | / | 1333  (60.43) | Self-Developed E-Health Literacy Questionnaire | 12.69±2.06 | (1)Well being (2)Psychological needs (3)Negative emotions | (1)General Well-Bing Schedule(GWB) (2)Basic Psychological Needs Scale(BPNS) (3)The Positive and Negative Affect Scale(PANAS） |
| [46] | [77] | Biscaldi V et al. (2023) | Italia | 614 | University students | 22.50±3.58 | 513  (83.6) | DHLI | 2.80±0.42 | (1)Well being (2)Health complaints | (1)WHO-5 Well-Being Index (2)The HBSC Symptom Checklist (HBSC-SCL) |
| [47] | [78] | Reitegger F et al. (2023) | Austria | 480 | Undergraduate a  Graduate students | Average:24.50 | 1624  (70.03 ) | Modified DHLI | / | (1)Well being (2)Future-Anxiety | (1)the World Health Organization (WHO) well-being scale (2)Future-Anxiety Scale |
| [48] | [79] | Xie C Y et al. (2020) | China | 1 115 | Undergraduate nursing students | 20.19±0.55 (18.00~22.00) | 1115  (100.0) | eHEALS | / | Depression | Epidemiological Investigation Center Depression Scale |
| [49] | [80] | Tran HTT et al. (2022) | Vietnam | 1851 | Undergraduate nursing students | 20.50 ± 1.20 | 1723(93.1) | eHEALS | 31.4 ± 4.4 | (1)Fear of COVID-19 (2)Anxiety (3)Depression (4)COVID-19 specific precautionary behaviors: Frequent hand washing (5)COVID-19 specific precautionary behaviors: Wearing a mask (6)COVID-19 specific precautionary behaviors: Physical distancing | (1)The seven-item fear of COVID-19 scale (2)seven-item Generalized Anxiety Disorder(GAD-7) (3)Nine-item Patient Health Questionnaire (PHQ-9) (4)(5)(6)Adapted from the existing literature |
| [50] | [81] | Wang Y et al. (2022) | China | 1085 | Undergraduate students | 20.01 ±1.44（17.00～25.00） | 401  （37.0） | eHEALS | 28.99±7.86 | (1)Health anxiety (2)Cyberchondria | (1)Chinese Version of the Short Health Anxiety Inventory (2)Chinese Version of the Short Cyberchondria Severity Scale |
| [51] | [82] | Ryan Michael F et al. (2021) | Filipino | 1,367 | undergraduate nursing students | 20.37 ± 2.85 | 1100  (80.5) | eHEALS | 3.88 ±0.61 | Fear of COVID-19 | The Fear of COVID-19 Scale (FCV-19S) |
| [52] | [83] | VÂJÂEan CC et al. (2015) | Romania | 513 | Undergraduate students | 23.24± 4.95 (19.00 ~ 35.00 ) | 413  (81.14 ) | eHEALS | / | (1)Compulsiveness with online health information seeking (2)Distress with online health information seeking (3)Online health information seeking behavior (4)Physical activity (5)Diet change | (1)(3)(4)(5)Self-designed items based on previous literature (2)Cyberchondria Severity Scale(CSS) |
| [53] | [84] | Amoako I et al. (2023) | Ghana | 1160 | Undergraduate students Graduate students | 26.88 ±5.53 | 325(28.0 ） | COVID-19 Digital Health literacy | / | Sense cf coherence | Sense of coherence -9 scale(SOC-9 scale) |
| [54] | [85] | Kim S et al. (2021) | South Korea | 558 | Nursing students | 20.30±2.20 (17.00～43.00) | 493  (88.4) | eHEALS | 30.53±5.20(15~40) | (1)Self-Care Agency (2)Social Media Use for Health Information (3)Online Health Information- Seeking Behaviors (4)Health-Promoting Behaviors | (1)Self-as-Carer Inventory (SCI)  (2)Self-designed items based on previous literature  (3)Online health information-seeking behavior instrument (4)Health Promoting Lifestyle Profile (HPLP-Ⅱ) |
| [55] | [86] | Paige SR et al.（2017） | America | 327 | Undergraduate students graduate students | 24.00±7.00 | 166  (50.8) | eHEALS | 29.49±5.71 (9-40 ) | （1）Online bridging social capital ability （2）Online bonding social capital ability （3）Social media use | Self-designed items based on previous literature |
| [56] | [87] | Zhong M et al.（2016） | China | 1 555 | Associate degree students Undergraduate students Graduate students | / | 1 062 ( 68.3) | eHEALS | 27.27±6.35 | Accessing and using electronic health information ability | Self-designed items based on the 2012 US Health Information Trends Scale |
| [57] | [88] | Hu J M et al. (2022) | China | 770 | Associate degree student Undergraduate students Graduate students PhD students | 21.59±2.65 (18.00~31.00) | 558  (72.47) | eHEALS | 32.00(28.00,32.00) | Capability of detecting online rumors during public health emergencies | Assessment of Online Rumor Discernment Ability During Public Health Emergencies |
| [58] | [89] | Yu Y et al. (2019) | China | 178 | University students | 20.00~39.00 | 82  (46.07) | Perceived e-health literacy (PEHL) | 4.84±0.937 | (1)Efficiency use of mobile healthcare applications (2)Effectiveness use of mobile healthcare applications | Self-designed items based on previous literature |
| [59] | [90] | Tong W et al. (2023) | China | 4621 | University students | 19.20±2.98 | 1918  (43.6) | eHEALS | Male: 28.55±7.94 Female: 29.52±7.15 | Mobile Phone Addiction | The Tendency to Mobile Phone Addiction Scale |
| [60] | [91] | Luo YF et al. (2018) | China(Taiwan) | 489 | College students | / | 306  (62.58) | EHLS | Functional eHealth literacy: 3.66±0.70 Interactive eHealth literacy: 3.67±0.67 Critical eHealth literacy: 3.65±0.69 | (1)Ability of making good use of various kinds of health care service organizations (2)Ability of making good use of a multitiered health care system (3)Ability of seeking medical advice based on different needs Frequency of medical use | Health Services Utilization Scale |
| [61] | [92] | Jiang L H et al. (2022) | China | 1446 | Undergraduate students | 19.78±1.60 | 1 237  (85.55) | eHEALS | 29.72±6.19 | Physical health | Adapted from the National Student Physical Health Standards (2014 Revision) |
| [62] | [93] | Park J W et al. （2017） | South Korea | 134 | Undergraduate students | 18.0~25.0 | 134  (100) | eHEALS | 3.42±0.61 | Self-care agency | Self-As-Carer Inventory |
| [63] | [94] | Hsu W et al. (2014) | China(Taiwan) | 525 | College students | / | / | EHLS | / | (1)Health Behavior (2)Regular eating habits (3)Physical activity habit (4)sleep habit | The Health Behavior Scale (HBS) |
| [64] | [95] | Ju-Young H et al. (2019) | Malaysia | 138 | Undergraduate nursing students | Mean age : 21.00 | 114  (82.6) | eHEALS | Female:3.78±0.58 Male:4.10±0.56 | Healthy Lifestyle Behavior | Health Promoting Life Style (HPLP) |
| [65] | [96] | Hwang A R et al. (2019) | South Korea | 242 | Undergraduate students | 21.64±1.85 | 137  (56.6) | EHL (eHealth Literacy) | 3.51±0.46 | Healthy Lifestyle Behavior | Health Promoting Lifestyle Profile (HPLP-II) |
| [66] | [97] | Kim KA et al.（2023） | South Korea | 358 | Undergraduate nursing students | 20.92 ±2.35（18.00~29.00) | 300  (83.8) | eHEALS | 30.08 ±5.07（11-40） | （1）Healthy lifestyle Behavior  （2）COVID‐19 related preventive behaviours （3）Satisfaction with major（1-5） （4）Satisfaction with campus life（1-5） （5）Time spent seeking health information online | (1)Health Promoting Lifestyle Profile‐II (HPLP‐II) (2)(3)(4)adapted from the existing literature |
| [67] | [98] | Li S J et al. (2022) | China | 2173 | Undergraduate students | 19.28 ± 1.20 （17.00~ 24.00） | 1144  (52.65) | eHEALS | Male:30.53±6.57 Female:30.13±6.04 | (1)Healthy lifestyle Behavior  (2)Physical activity  (3)Regular lifestyle (4)Nutrition (5)Maintain lifestyle free of harmful substances (6)Health responsibility behaviors for maintaining personal and public hygiene (7)Interpersonal relationships (8)Stress management (9)Life satisfaction and positive growth behaviors | Modified health lifestyle scale for college students |
| [68] | [99] | Cui G H et al. (2020) | China | 1442 | Undergraduate medicine students | 18.19±0.58 (17.00～22.00) | 1 052  (72.95) | The eHealth Literacy Scale for College Students(20–100) | 73.62±14.95 | (1)Healthy lifestyle Behavior  (2)Physical activity  (3)Regular lifestyle (4)Nutrition (5)Maintain lifestyle free of harmful substances (6)Health responsibility behaviors for maintaining personal and public hygiene (7)Interpersonal relationships (8)Stress management (9)Life satisfaction and positive growth behaviors | Modified health lifestyle scale for college students |
| [69] | [100] | Wu Q et al. (2022) | China | 2640 | Undergraduate students | 19.71±1.31 (17.00～24.00 ) | 1629 ( 61.7) | The eHealth Literacy Scale for College Students | Male:76.65±17.08 Female:73.72±15.83 | (1)Healthy lifestyle Behavior  (2)Physical activity  (3)Regular lifestyle (4)Nutrition (5)Maintain lifestyle free of harmful substances (6)Health responsibility behaviors for maintaining personal and public hygiene (7)Interpersonal relationships (8)Stress management (9)Life satisfaction and positive growth behaviors | Modified health lifestyle scale for college students |
| [70] | [101] | Kasımoğlu N et al. (2023) | Turkey | 1,714 | Undergraduate students | 21.03±2.27 | 1097  (64.0) | eHEALS | 27.80±6.12 | (1)Health responsibility behaviors for maintaining personal and public hygiene (2)Physical Activity (3)Nutrition (4)Mental Development (5)Interpersonal Relationships (6)Stress Management | The Healthy Lifestyle Behaviors Scale II |
| [71] | [102] | Eyimaya A et al. (2021) | Turkey | 390 | Undergraduate students | / | 329  （84.4） | eHEALS | Male: 28.31 ± 5.04 Female: 27.37±4.61 | Healthy Lifestyle Behavior | Healthy Lifestyle Behavior Scale II (HLBS II) |
| [72] | [103] | Wang S S et al. (2015) | China | 984 | Associate degree students Undergraduate students Graduate students PhD students | 21.32±2.18 | 411  （41.8） | eHEALS | 26.58±5.77 | (1)Healthy lifestyle Behavior  (2)Physical activity (3)Balanced dietary behavior (4)Sufficient sleep (5)Smoking habit (6)Alcohol habit | Self-designed items based on previous literature |
| [73] | [104] | Lee S M et al. (2018) | South Korea | 306 | Undergraduate health science students | / | 258  (84.3) | eHEALS | 3.59±0.57 | Healthy lifestyle Behavior | HBS(Health Behavior Scale) |
| [74] | [105] | Öztürk E et al. (2023) | Turkey | 279 | Undergraduate nursing students | / | 193  ( 69.2) | eHEALS | 28.97±6.324 | Healthy lifestyle Behavior | Health Promotion and Protective Behaviors Scale (HPPBS) |
| [75] | [106] | Meng S X et al. （2018） | China | 1023 | Undergraduate students | / | 445（43.50） | eHEALS | 27.42 ±5.31 | (1)Staying up late (2)Smoking | Self-designed items based on previous literature |
| [76] | [107] | Tian H et al. (2022) | China | 5,151 | University students | 18.00~22.00 | 3680  (71.46) | eHEALS | 26.81 ±5.83 | (1)Physical activity (2)Regular breakfast behavior (3)Smoking (4)Alcohol (5)Sufficient sleep | Self-designed items based on previous literature |
| [77] | [108] | [Tariq A et al. (2020](https://pubmed.ncbi.nlm.nih.gov/?term=Tariq A[Author])) | Pakistan | 900 | Undergraduate degree  Postgraduate | ≥16.00 | 211  (41.8) | eHEALS | Median score: 29 | (1)Physical activity behavior (2)Nutrition | Self-designed items based on previous literature |
| [78] | [109] | Huang CL et al. (2020) | China(Taiwan) | 674 | College Students | 20.44±2.03 | 286  (42.43) | EHLS | Functional：3.94 ±0.77 Interactive:3.66 ±0.74 Critical: 3.78±0.79 | (1)Dietary behavior (2)Physical activity | Adapted items based on previous literature |
| [79] | [110] | Lee B C et al. (2021) | South Korea | 191 | Undergraduate students | / | 66  (34.6) | Modified eHEALS | / | (1)Dietary behavior (2)Sufficient sleep (3)Physical activity | Self-designed items based on previous literature |
| [80] | [111] | Yang SC et al. (2019) | China(Taiwan) | 813 | Colleges | 20.08± 1.43 | 383  (47.1) | EHLS | Functional eHealth literacy: 3.56±0.77  Interactive eHealth literacy: 3.57±0.71  Critical eHealth literacy:3.59 ± 0.72 | (1)Unhealthy food intake (2)Balanced diet (3)Health consumption pattern (4)Regular eating habits | Dietary Behaviors Scale |
| [81] | [112] | Acar AK et al. (2021) | Turkey | 535 | Undergraduate students | 18.00~25.00 | 401  (74.95) | eHEALS | / | (3)Physical Activity | International Physical Activity Questionnaire ShortForm (IPAQ-SF), |
| [82] | [113] | Williams MSZ et al. （2014） | America | 160 | Undergraduate students Graduate students | 19.00~29.00 | 130  (81.3) | Modified eHEALS (0-50) | 40.4±5.2 ( 24 - 50) HPV vaccinated: 39.34 ± 5.37; Unvaccinated: 41.15 ± 4.90 | HPV vaccine uptake | Self-designed items based on previous literature |
| [83] | [114] | Hong KJ et al. (2021) | South Korea | 274 | Healthcare undergraduates (nursing, pathology, occupational therapy) | / | 237  (86.5) | e-HL tool | Male: 3.74 ± 0.62 Female: 3.61 ± 0.60 | COVID‐19 related preventive behaviors | Tool developed by Lee et al.based on the Prevention Guideline on Droplet Infection |
| [84] | [115] | Hadley MK( 2022) | America | 245 | Undergraduate students | 20.90± 4.20 | 196  （80.0） | Modified DHLI | 18±4.3( 9-36) | (1)COVID-19-specific precautionary behaviors: Frequent hand washing (2)COVID-19-specific precautionary behaviors: Physical distancing (3)COVID-19-specific precautionary behaviors: Wearing a mask (4)COVID-19-specific precautionary behaviors: Staying at home and only leaving for essential trips | Self-designed items based on previous literature |
| [85] | [116] | Jiang X X et al. (2023) | China | 788 | Undergraduate students Graduate students PhD students | ≥17.00 | 390  (49.49) | eHEALS | Chinese:30.28±6.56 Korean: 29.48±7.0 | (1)COVID-19-specific precautionary behaviors: Wearing a mask (2)COVID-19-specific precautionary behaviors: Physical distancing (3)COVID-19-specific precautionary behaviors: Avoiding crowded places (4)COVID-19-specific precautionary behaviors: Regular indoor ventilation | Adapted from the existing literature |
| [86] | [117] | Liu J C et al. (2020) | China | 1 157 | Vocational students | 19.13±0.93  (17.00~23.00) | 690  (59.64) | eHEALS | 29. 92±8. 36 | Disease coping behavior | Disease Behavior Assessment Scale |
| [87] | [118] | Luo L et al. (2021) | China | 2332 | Undergraduate degree  Postgraduate | 18.00～30.00 | 2332  (100.0) | eHEALS | 27.17±5.85 | Dysmenorrhea Management Behavior | Dysmenorrhea Management Questionnaire |
| [88] | [119] | Göde A et al. (2023) | Turkey | 542 | Vocational students | ≥18.00 | 377  ( 69.6) | eHEALS | 3.56±0.69 | Rational Drug Use | Rational Drug Use Scale |
| [89] | [120] | Kaynak S et al. (2022) | Turkey | 341 | Undergraduate nursing students | 21.23±1.82 | 256  (75.1) | eHEALS | 29.24±5.02 | Clinical decision making | The clinical decision making in nursing scale |

eHEALS: e-Health Literacy Scale; EHLS: eHealth Literacy Scale; DHLI: Digital Health Literacy Instrument

**References**

1. Yuan T, Liu H, Li XD, Liu HR: **Factors Affecting Infection Control Behaviors to Prevent COVID-19: An Online Survey of Nursing Students in Anhui, China in March and April 2020**. *Medical science monitor : international medical journal of experimental and clinical research* 2020, **26**:e925877.

2. Yang SC, Luo YF, Chiang CH: **The Associations Among Individual Factors, eHealth Literacy, and Health-Promoting Lifestyles Among College Students**. *Journal of medical Internet research* 2017, **19**(1):e15.

3. Patil U, Kostareva U, Hadley M, Manganello JA, Okan O, Dadaczynski K, Massey PM, Agner J, Sentell T: **Health Literacy, Digital Health Literacy, and COVID-19 Pandemic Attitudes and Behaviors in U.S. College Students: Implications for Interventions**. *International journal of environmental research and public health* 2021, **18**(6).

4. Tsukahara S, Yamaguchi S, Igarashi F, Uruma R, Ikuina N, Iwakura K, Koizumi K, Sato Y: **Association of eHealth Literacy With Lifestyle Behaviors in University Students: Questionnaire-Based Cross-Sectional Study**. *Journal of medical Internet research* 2020, **22**(6):e18155.

5. Lotto M, Maschio KF, Silva KK, Ayala Aguirre PE, Cruvinel A, Cruvinel T: **eHEALS as a predictive factor of digital health information seeking behavior among Brazilian undergraduate students**. *Health Promot Int* 2023, **38**(4).

6. Dallora AL, Andersson EK, Gregory Palm B, Bohman D, Björling G, Marcinowicz L, Stjernberg L, Anderberg P: **Nursing Students' Attitudes Toward Technology: Multicenter Cross-Sectional Study**. *JMIR Med Educ* 2024, **10**:e50297.

7. Oducado RMJAoTM, Health P: **Filipino nursing students' ehealth literacy and criteria used for selection of health websites**. 2020, **23**.

8. Qin N, Shi S, Ma G, Li X, Duan Y, Shen Z, Luo A, Zhong Z: **Associations of COVID-19 Risk Perception, eHealth Literacy, and Protective Behaviors Among Chinese College Students Following Vaccination: A Cross-Sectional Study**. *Frontiers in public health* 2021, **9**:776829.

9. Li S, Cui G, Kaminga AC, Cheng S, Xu H: **Associations Between Health Literacy, eHealth Literacy, and COVID-19-Related Health Behaviors Among Chinese College Students: Cross-sectional Online Study**. *Journal of medical Internet research* 2021, **23**(5):e25600.

10. Qin N, Shi S, Duan Y, Ma G, Li X, Shen Z, Zhang S, Luo A, Zhong Z: **Social Media Use, eHealth Literacy, Knowledge, Attitudes, and Practices Toward COVID-19 Vaccination Among Chinese College Students in the Phase of Regular Epidemic Prevention and Control: A Cross-Sectional Survey**. *Frontiers in public health* 2021, **9**:754904.

11. Mai, J. R., Zhou, L., He, J. N., Huang, T. F., & Lin, L. N. Correlative Analysis of E-Health Literacy and Infectious Disease Health Literacy Among Nursing Undergraduates in Guangdong Province [J]. Chinese Nursing Education, 2022, 19(08): 719-722. DOI: 10.3761/j.issn.1672-9234.2022.08.010

12. Aslantekin-Özcoban F, Gün MJC, Obstetrics E, Gynecology: **Emergency contraception knowledge level and e-health literacy in Turkish university students**. 2021, **48**(6):1424-1431.

13. Kılınç İşleyen E, Korkmaz Aslan G, Kartal A: **Knowledge and Perceptions About Cervical Cancer and Human Papillomavirus, and Relationship with E-health Literacy, and Affecting Factors Among Female University Students**. *Journal of adolescent and young adult oncology* 2024, **13**(3):564-572.

14. Zhang S, Wang W, Wu S, Ye H, Dong L, Wang J, Ning X, Cui H: **Analysis of the mediating effect between ehealth literacy and health self-management of undergraduate nursing students' mental health literacy**. *BMC nursing* 2024, **23**(1):264.

15. Mayukh NJJoC, Language, Culture: **The Influence of eHealth Literacy and Self-Efficacy on Online Health Information-Seeking Behaviour among University Students: Cyberchondria as a Mediator**. 2024, **4**(1):40-60.

16. Bao, X. L. Research on the influence of college students' epidemic prevention and control cognition on their healthy lifestyle [Doctoral dissertation, Southern Medical University]. 2022. DOI: 10.27003/d.cnki.gojyu.2022.001045

17. Sögüt S, Cangöl E, Dolu İ: **The Relationship Between eHealth Literacy and Self-Efficacy Levels in Midwifery Students Receiving Distance Education During the COVID-19 Pandemic**. *J Nurs Res* 2022, **30**(2):e203.

18. Sun H, Qian L, Xue M, Zhou T, Qu J, Zhou J, Qu J, Ji S, Bu Y, Hu Y *et al*: **The relationship between eHealth literacy, social media self-efficacy and health communication intention among Chinese nursing undergraduates: A cross-sectional study**. *Frontiers in public health* 2022, **10**:1030887.

19. Turan N, Güven Özdemir N, Çulha Y, Özdemir Aydın G, Kaya H, Aştı T: **The effect of undergraduate nursing students' e-Health literacy on healthy lifestyle behaviour**. *Glob Health Promot* 2021, **28**(3):6-13.

20. Pisl V, Volavka J, Chvojkova E, Cechova K, Kavalirova G, Vevera J: **Dissociation, Cognitive Reflection and Health Literacy Have a Modest Effect on Belief in Conspiracy Theories about COVID-19**. *International journal of environmental research and public health* 2021, **18**(10).

21. Zadeh Kh., Ghazavi, Salamat A. J. Health Literacy: Investigating the Relationship Between Health Literacy, Conspiracy Beliefs, and Future Anxiety in the Face of COVID-19. Journal Name, 2023, 20(1), 50–55. DOI: 10.48305/him.2023.41589.1099

22. Chen, Y. N. A study on cervical cancer information-seeking behavior among female college students [Master’s thesis, Sichuan International Studies University]. 2023.DOI: 10.27348/d.cnki.gscwc.2023.000269.

23. Chun, H. R., Yoon, H. R., & Choi, S. G., Korean Journal of Population Studies: Digital health literacy and preventive health behaviors among college students: Focusing on COVID-19 vaccination intention and participation in preventive measures. 2021, 44(2):121-141.DOI: 10.31693/KJPS.2021.06.44.2.121

24. Pisl V, Volavka J, Chvojkova E, Cechova K, Kavalirova G, Vevera J: **Willingness to Vaccinate Against COVID-19: The Role of Health Locus of Control and Conspiracy Theories**. *Frontiers in psychology* 2021, **12**:717960.

25. Kıbrıs Ş, Kızılkaya SJSvSRAD: **E-SAĞLIK OKURYAZARLIK DÜZEYİNİN SAĞLIK ALGISI ÜZERİNE ETKİSİNİN İNCELENMESİ**. 2023, **5**(2):241-250.

26. Liao LL, Chang LC, Lai IJ, Lee CK: **College Students' E-health Literacy, Social Media Use, and Perceptions of E-cigarettes in Taiwan**. *J Community Health* 2024, **49**(1):52-60.

27. Fehér A, Véha M, Boros HM, Kovács B, Kontor E, Szakály Z: **The Relationship between Online and Offline Information-Seeking Behaviors for Healthy Nutrition**. *International journal of environmental research and public health* 2021, **18**(19).

28. Noh M Y. The effect of e-health literacy on exercise self-schema among female college students in vocational schools. Journal of the Korean Association of Physical Education and Sport for Girls and Women, 2021, 35(1), 85-97.DOI : 10.16915/jkapesgw.2021.3.35.1.85

29. Britt RK, Collins WB, Wilson K, Linnemeier G, Englebert AM: **eHealth Literacy and Health Behaviors Affecting Modern College Students: A Pilot Study of Issues Identified by the American College Health Association**. *Journal of medical Internet research* 2017, **19**(12):e392.

30. Yan X D. Exploring the Mechanism of Effectively Using Mobile Healthcare Applications [D]. Tianjin University, 2018.

31. Wang X, Yue T, Mo PK: **The associations among cognitive social factors, eHealth literacy and health-promoting behaviors in Chinese adolescents**. *Health Promot Int* 2022, **37**(6).

32. Chen SC, Huy LD, Lin CY, Lai CF, Nguyen NTH, Hoang NY, Nguyen TTP, Dang LT, Truong NLT, Phan TN *et al*: **Association of Digital Health Literacy with Future Anxiety as Mediated by Information Satisfaction and Fear of COVID-19: A Pathway Analysis among Taiwanese Students**. *International journal of environmental research and public health* 2022, **19**(23).

33. Chen SC, Hong Nguyen NT, Lin CY, Huy LD, Lai CF, Dang LT, Truong NLT, Hoang NY, Nguyen TTP, Phaṇ TN *et al*: **Digital health literacy and well-being among university students: Mediating roles of fear of COVID-19, information satisfaction, and internet information search**. *Digital health* 2023, **9**:20552076231165970.

34. Kim J O. The Reliability of Health Information on the Internet and the Medical Advertising’s Attitude on the Internet according to e-Health Literacy Level. Humanities and Social Sciences, 2017, 8(4):299-314.

35. Nam Y H. A comparative study of e-health literacy, health information credibility, and health behaviors affecting health information usage motivation between Korean and Chinese university students. Journal of Digital Contents Society, 2020, 21(3):513-520. DOI : 10.9728/dcs.2020.21.3.513

36. Masilamani V, Arulchelvan S, Rozario AMJCRCdCyE: **Alfabetización en e-Salud de los jóvenes: Credibilidad y calidad de la información sanitaria con móviles en la India**. 2020(64):85-95.

37. Kim H S, Lee K H. A study on perceptions and activation strategies of community service activities among health sciences university students. The Journal of the Korea Academia-Industrial Cooperation Society, 2021, 22(10):304-315. DOI : 10.5762/KAIS.2021.22.10.304

38. Kuang H D, Li J, Gu Z J, et al. The mediating effect of e-health literacy between mental health and online psychological help-seeking behavior among college students [J]. China Journal of Health Psychology, 2023, 31(12): 1876-1880. DOI:10.13342/j.cnki.cjhp.2023.12.022.

39. Amoah PA, Leung AYM, Parial LL, Poon ACY, Tong HH, Ng WI, Li X, Wong EML, Kor PPK, Molassiotis A: **Digital Health Literacy and Health-Related Well-Being Amid the COVID-19 Pandemic: The Role of Socioeconomic Status Among University Students in Hong Kong and Macao**. *Asia-Pacific journal of public health* 2021, **33**(5):613-616.

40. Chen W, Zheng Q, Liang C, Xie Y, Gu D: **Factors Influencing College Students' Mental Health Promotion: The Mediating Effect of Online Mental Health Information Seeking**. *International journal of environmental research and public health* 2020, **17**(13).

41. Xu G, Xu Y, Tu X, Hao S, Liu T: **The Association between Self-Rated Health and Health Self-Management Ability of Healthcare Undergraduates: The Chain Mediating Roles of eHealth Literacy and Resistance to Peer Influence**. *International journal of environmental research and public health* 2022, **19**(21).

42. Rivadeneira MF, Miranda-Velasco MJ, Arroyo HV, Caicedo-Gallardo JD, Salvador-Pinos C: **Digital Health Literacy Related to COVID-19: Validation and Implementation of a Questionnaire in Hispanic University Students**. *International journal of environmental research and public health* 2022, **19**(7).

43. Choi S: **Comparison of Self-Tracking Health Practices, eHealth Literacy, and Subjective Well-Being Between College Students With and Without Disabilities: Cross-Sectional Survey**. *JMIR formative research* 2024, **8**:e48783.

44. Rivadeneira MF, Salvador C, Araujo L, Caicedo-Gallardo JD, Cóndor J, Torres-Castillo AL, Miranda-Velasco MJ, Dadaczynski K, Okan O: **Digital health literacy and subjective wellbeing in the context of COVID-19: A cross-sectional study among university students in Ecuador**. *Frontiers in public health* 2022, **10**:1052423.

45. Ha L N, Chang Q N, Chen X. The impact of e-health literacy on well-being in medical students: A serial mediation model of basic psychological needs and negative emotions [J]. China Journal of Health Psychology, 2023, 31(9): 1381-1388. DOI: 10.13342/j.cnki.cjhp.2023.09.019

46. Biscaldi V, Delbosq S, Ghelfi M, Serio J, Vecchio L, Dadaczynski K, Okan O, Velasco V: **A cross-sectional study of university students' wellbeing: What to focus on?** *PSICOLOGIA DELLA SALUTE* 2023:105-124.

47. Reitegger F, Wright M, Berger J, Gasteiger-Klicpera B: **Digitale Gesundheitskompetenz und Wohlbefinden**. *Prävention und Gesundheitsförderung* 2023, **18**(2):204-210.

48. Xie C Y, Li S J, Hu J Y. Association between e-health literacy, social support and depressive symptoms among female nursing students [J]. Chinese Journal of School Health, 2020, 41(5): 716-719. DOI: 10.16835/j.cnki.1000-9817.2020.05.022.

49. Tran HTT, Nguyen MH, Pham TTM, Kim GB, Nguyen HT, Nguyen NM, Dam HTB, Duong TH, Nguyen YH, Do TT *et al*: **Predictors of eHealth Literacy and Its Associations with Preventive Behaviors, Fear of COVID-19, Anxiety, and Depression among Undergraduate Nursing Students: A Cross-Sectional Survey**. *International journal of environmental research and public health* 2022, **19**(7).

50. Wang Y. The influence of e-health literacy and health anxiety on cyberchondria among university students [D]. Yanbian University, 2022. DOI: 10.27439/d.cnki.gybdu.2022.000603.

51. Ryan Michael F, Tuppal CP, Estoque HV, Sadang JM, Superio DL, Don Vicente C, Mary Nellie T, Xerxes G, Quiros JD, Fajardo MTR: **Uso de Internet y la alfabetización en eSalud con temor al COVID-19 entre estudiantes de enfermería en Filipinas Internet use, eHealth literacy and fear of COVID-19 among nursing students in the Philippines**.

52. VÂJÂEan CC, BĂBan A: **EMOTIONAL AND BEHAVIORAL CONSEQUENCES OF ONLINE HEALTH INFORMATION-SEEKING: THE ROLE OF EHEALTH LITERACY**. *Cognitie, Creier, Comportament/Cognition, Brain, Behavior* 2015, **19**(4):327-345.

53. Amoako I, Srem-Sai M, Quansah F, Anin S, Agormedah EK, Hagan Jnr JE: **Moderation modelling of COVID-19 digital health literacy and sense of coherence across subjective social class and age among university students in Ghana**. *BMC Psychol* 2023, **11**(1):337.

54. Kim S, Oh J: **The Relationship between E-Health Literacy and Health-Promoting Behaviors in Nursing Students: A Multiple Mediation Model**. *Int J Environ Res Public Health* 2021, **18**(11).

55. Paige SR, Stellefson M, Chaney BH, Chaney JD, Alber JM, Chappell C, Barry AE: **Examining the Relationship between Online Social Capital and eHealth Literacy: Implications for Instagram Use for Chronic Disease Prevention among College Students**. *Am J Health Educ* 2017, **48**(4):264-277.

56. , Xu X Y. Association between individual factors, e-health literacy and health information utilization among university students in Guangzhou [J]. Chinese Journal of School Health, 2016, 37(12): 1787-1790. DOI: 10.16835/j.cnki.1000-9817.2016.12.009

57. Hu J M, Li H L, Yang Y L, Zhang Y W, He X F, Shi L. Investigation of college students' ability to identify online rumors during public health emergencies [J]. Journal of Nursing Science, 2022, 37(8): 65-68+93. DOI: 10.3870/j.issn.1001-4152.2022.08.065

58. Yu Y, Yan X, Zhang X, Zhou S: **What they gain depends on what they do: an exploratory empirical research on effective use of mobile healthcare applications**. 2019.

59. Tong W, Meng S: **Effects of Physical Activity on Mobile Phone Addiction Among College Students: The Chain-Based Mediating Role of Negative Emotion and E-Health Literacy**. *Psychol Res Behav Manag* 2023, **16**:3647-3657.

60. Luo YF, Yang SC, Chen AS, Chiang CH: **Associations of eHealth Literacy With Health Services Utilization Among College Students: Cross-Sectional Study**. *Journal of medical Internet research* 2018, **20**(10):e283.

61. Jiang L H, Guo X Y, Lu B Y, et al. Correlation between e-health literacy and physical health among college students [J]. Chinese Journal of School Health, 2022, 43(7): 990-994. DOI: 10.16835/j.cnki.1000-9817.2022.07.008

62. Park J W. A comparative study of e-health literacy and self-care competence between nursing students and non-health major female college students [J]. Journal of Korean Academy of Nursing Administration, 2017, 23(4): 439-449. DOI: 10.11111/jkana.2017.23.4.439.

63. Hsu W, Chiang C, Yang S: **The effect of individual factors on health behaviors among college students: the mediating effects of eHealth literacy**. *Journal of medical Internet research* 2014, **16**(12):e287.

64. Ju-Young H, Lee S-YJM-LU: **The relationship between the subjective health status, e-Health literacy, health literacy and health promoting behavior in under graduate nursing students**. 2019, **19**(1).

65. Hwang A R, Je J J. The influence of university students' e-health literacy on health promotion behaviors[J]. 2019, 32(3). DOI: 10.15434/kssh.2019.32.3.165

66. Kim KA, Hyun MS, De Gagne JC, Ahn JA: **A cross-sectional study of nursing students' eHealth literacy and COVID-19 preventive behaviours**. *Nursing open* 2023, **10**(2):544-551.

67. Li S J, Cui G H, Xu H L. Path analysis of internet social support, e-health literacy and health-related behaviors among college students [J]. Chinese Journal of Health Statistics, 2022, 39(1): 118-121. DOI: 10.3969/j.issn.1002-3674.2022.01.027.

68. Cui G H, Yin Y T, Wang M Z, et al. The relationship between e-health literacy and healthy lifestyles among medical students [J]. Chinese Journal of School Health, 2020, 41(6): 936-938. DOI: 10.16835/j.cnki.1000-9817.2020.06.037.

69. Wu Q, Zhao G H, Gong J, et al. Status and correlation analysis of e-health literacy and healthy lifestyles among university students in Wuhan [J]. Medicine and Society, 2022, 35(8): 78-83. DOI: 10.13723/j.yxysh.2022.08.015.

70. Kasımoğlu N, Karakurt P, Başkan SAJIJOHSR, Policy: **THE RELATIONSHIP BETWEEN UNIVERSITY STUDENTS’E-HEALTH LITERACY AND HEALTHY LIFESTYLE BEHAVIORS**. 2023, **8**(1):38-47.

71. Eyimaya A, Özdemir F, Tezel A, Apay SE: **Determining the healthy lifestyle behaviors and e-health literacy levels in adolescents**. *Revista da Escola de Enfermagem da U S P* 2021, **55**:e03742.

72. WWang S S. Research on the eHealth literacy of college students in Hangzhou [D]. Hangzhou Normal University, 2015.

73. Lee S M. The Effect of e-Health literacy on Health Behavior in Health Science Majors [J]. The Journal of Korean Society for School & Community Health Education, 2018, 19(2): 77-86.

74. Öztürk E, Işık SS, Can ZJHSHD: **Determining the Relationship Between e-Health Literacy and Health-Improving and Protective Behaviors in Nursing Students**. **5**(2):106-116.

75. Meng S X, Shen C. Investigation on e-health literacy and behavior status among university students in Nanjing[J]. Chinese Journal of Health Education, 2018, 34(3): 254-257. DOI: 10.16168/j.cnki.issn.1002-9982.2018.03.014

76. Tian H, Chen J: **The association and intervention effect between eHealth literacy and lifestyle behaviors among Chinese university students**. *Revista da Escola de Enfermagem da U S P* 2022, **56**:e20220147.

77. Tariq A, Khan SR, Basharat A: **Internet Use, eHealth Literacy, and Dietary Supplement Use Among Young Adults in Pakistan: Cross-Sectional Study**. *Journal of medical Internet research* 2020, **22**(6):e17014.

78. Huang CL, Yang SC, Chiang CH: **The Associations between Individual Factors, eHealth Literacy, and Health Behaviors among College Students**. *International journal of environmental research and public health* 2020, **17**(6).

79. Lee B C. The relationship between e-health literacy and health behaviors among university students [J]. Journal of Convergence for Sport Science, 2021, 19(2): 55-62. DOI: 10.22997/jcses.2021.19.2.55.

80. Yang SC, Luo YF, Chiang CH: **Electronic Health Literacy and Dietary Behaviors in Taiwanese College Students: Cross-Sectional Study**. *Journal of medical Internet research* 2019, **21**(11):e13140.

81. Acar AK, Savcı S, Kahraman BÖ, Tanrıverdi AJJoB, Sciences CH: **Comparison of E-Health Literacy, Digital Health and Physical Activity Levels Of University Students In Different Fields**. 2021, **8**(2):380-389.

82. Williams MSZ: **A mixed methods study of health literacy and its role in hpv vaccine uptake among college students**; 2014.

83. Hong KJ, Park NL, Heo SY, Jung SH, Lee YB, Hwang JH: **Effect of e-Health Literacy on COVID-19 Infection-Preventive Behaviors of Undergraduate Students Majoring in Healthcare**. *Healthcare (Basel, Switzerland)* 2021, **9**(5).

84. Hadley MK: **COVID-19 and digital health literacy in university students/narrative competence and cognitive mapping as a culturally sustaining pedagogy in the education of emergent bilinguals**. 2022.

85. Jiang X X. Prevention Behavior and Influencing Factors of COVID-19 [D]. Shandong University, 2023.

86. Liu J C, Yin Y T, Fan Y Y. Relationship between eHealth literacy and illness behavior among vocational college students in Jinan City[J]. Chinese Journal of School Health, 2020, 41(10): 1502-1505+1510. DOI: 10.16835/j.cnki.1000-9817.2020.10.016.

87. Luo L, Song N Q, Yuan J F, et al. Relationship between electronic health literacy and dysmenorrhea management behavior of female college students in Guizhou universities [J]. Modern Preventive Medicine, 2021, 48(23):4317-4323,4330.

88. Göde A, Öztürk YE, Kuşcu FNJJoIHS, Management: **Examining The Relationship Between E-Health Literacy and Rational Drug Use: A Study on University Students**. 2023, **9**(18):8-16.

89. Kaynak S, Arat N, Yardımcı F, Şenol S, Yılmaz HBJEÜHFD: **Hemşirelik öğrencilerinin E-sağlık okuryazarlık düzeyi ile klinik karar verme becerileri arasındaki ilişki**. 2022, **38**(3):229-237.
