# Supplementary material for: eHealth Literacy and Its Outcomes Among Postsecondary Students: Systematic Review
Source: J Med Internet Res. 2025 Jul 2;27:e64489. doi: 10.2196/64489 (PMC12278882; doi:10.2196/64489)
Supplement: Multimedia Appendix 4 [file jmir_v27i1e64489_app4.doc]

**Multimedia Appendix 4.** Risk of bias assessment of the 89 included studies.

| Citation in this file |  | Introduction | | Methods | | | | | | | | | |
| --- | --- | --- | --- | --- | --- | --- | --- | --- | --- | --- | --- | --- | --- |
|  | Citation corresponding to the manuscript reference | Author(year of publication) | 1.Were the aims/objectives of the study clear? | 2.Was the study design appropriate for the stated aim(s)? | 3.Was the sample size justified? | 4.Was the target/reference population clearly defined? (Is it clear who the research was about?) | 5.Was the sample frame taken from an appropriate population base so that it closely represented the target/reference population under investigation? | 6.Was the selection process likely to select subjects/participants that were representative of the target/reference population under investigation? | 7.Were measures undertaken to address and categorise non-responders? | 8.Were the risk factor and outcome variables measured appropriate to the aims of the study? | 9.Were the risk factor and outcome variables measured correctly using instruments/measurements that had been trialled, piloted or published previously? | 10.Is it clear what was used to determined statistical significance and/or precision estimates? (eg, p values, CIs) | 11.Were the methods (including statistical methods) sufficiently described to enable them to be repeated? |
| [1] | [19] | Yuan T, et al. (2020) | Y | Y | N | Y | Y | Y | N | Y | Y | Y | Y |
| [2] | [20] | Yang SC et al. (2017) | Y | Y | N | Y | Y | Y | Y | Y | Y | Y | Y |
| [3] | [21] | Patil U et al. (2021) | Y | Y | Y | Y | Y | Y | Y | Y | Y | Y | Y |
| [4] | [26] | Tsukahara S et al. (2020) | Y | Y | N | Y | Y | Y | N | Y | Y | Y | Y |
| [5] | [28] | Lotto M et al. (2023) | Y | Y | N | Y | Y | Y | N | Y | Y | Y | Y |
| [6] | [29] | [Ana Luiza Dallora et al. (2024](https://pubmed.ncbi.nlm.nih.gov/?term=Dallora AL[Author])) | Y | Y | N | Y | Y | Y | Y | Y | Y | Y | Y |
| [7] | [38] | Oducado et al. (2020) | Y | Y | Y | Y | Y | Y | N | Y | Y | Y | Y |
| [8] | [39] | Qin N et al. (2021) | Y | Y | N | Y | Y | Y | N | Y | Y | Y | Y |
| [9] | [40] | Li S et al. (2021) | Y | Y | N | Y | Y | Y | N | Y | Y | Y | Y |
| [10] | [41] | Qin N et al. (2021) | Y | Y | N | Y | Y | Y | N | Y | Y | Y | Y |
| [11] | [42] | Mai, J. R et al. （2022） | Y | Y | N | Y | Y | Y | N | Y | Y | Y | Y |
| [12] | [43] | Aslantekin-Özcoban F et al. (2021) | Y | Y | Y | Y | Y | Y | N | Y | Y | Y | Y |
| [13] | [44] | Kılınç İşleyen E et al. (2024) | Y | Y | Y | Y | N | N | N | Y | Y | Y | Y |
| [14] | [45] | Zhang S et al. (2024) | Y | Y | Y | Y | Y | Y | N | Y | Y | Y | Y |
| [15] | [46] | Mayukh NJJoC (2024) | Y | Y | N | Y | Y | Y | N | Y | Y | Y | Y |
| [16] | [47] | Bao, X. L et al. (2022) | Y | Y | N | Y | Y | Y | N | Y | Y | Y | Y |
| [17] | [48] | Sögüt S et al. (2022) | Y | Y | N | Y | Y | Y | N | Y | Y | Y | Y |
| [18] | [49] | Sun H(2022) | Y | Y | Y | Y | Y | Y | N | Y | Y | Y | Y |
| [19] | [50] | Turan N et al. (2021) | Y | Y | N | Y | Y | Y | N | Y | Y | Y | Y |
| [20] | [51] | Pisl V et al. (2021) | Y | Y | N | Y | N | N | N | Y | Y | Y | Y |
| [21] | [52] | Zadeh Kh et al. (2023) | Y | Y | Y | Y | Y | Y | N | Y | Y | Y | Y |
| [22] | [53] | Chen, Y. N et al. （2023） | Y | Y | N | Y | Y | Y | N | Y | Y | Y | Y |
| [23] | [54] | Chun, H. R et al. (2021) | Y | Y | N | Y | Y | Y | N | Y | Y | Y | Y |
| [24] | [55] | Pisl V et al. (2021) | Y | Y | N | Y | N | N | N | Y | Y | Y | Y |
| [25] | [56] | Kıbrıs Ş et al. (2023) | Y | Y | Y | Y | Y | Y | N | Y | Y | Y | Y |
| [26] | [57] | Liao LL et al. (2024) | Y | Y | N | Y | Y | Y | Y | Y | Y | Y | Y |
| [27] | [58] | Fehér A et al. (2021) | Y | Y | N | Y | Y | N | N | Y | Y | Y | Y |
| [28] | [59] | Noh M Y (2021) | Y | Y | N | Y | Y | Y | N | Y | Y | Y | Y |
| [29] | [60] | Britt RK et al. (2017) | Y | Y | N | Y | Y | Y | N | Y | Y | Y | Y |
| [30] | [61] | Yan X D et al. （2018） | Y | Y | N | Y | Y | Y | N | Y | Y | Y | Y |
| [31] | [62] | Wang X et al. (2022) | Y | Y | N | Y | Y | Y | N | Y | Y | Y | Y |
| [32] | [63] | Chen SC et al. （2022） | Y | Y | N | Y | Y | Y | N | Y | Y | Y | Y |
| [33] | [64] | Chen SC et al. (2023) | Y | Y | N | Y | Y | Y | N | Y | Y | Y | Y |
| [34] | [65] | Kim J O (2017) | Y | Y | Y | Y | Y | Y | N | Y | Y | Y | Y |
| [35] | [66] | Nam Y H et al. (2020) | Y | Y | N | Y | Y | Y | N | Y | Y | Y | Y |
| [36] | [67] | Masilamani V et al. （2020） | Y | Y | N | Y | Y | Y | N | Y | Y | Y | Y |
| [37] | [68] | Kim H S et al. (2021) | Y | Y | Y | Y | Y | Y | N | Y | Y | Y | Y |
| [38] | [69] | Kuang H D et al. （2023） | Y | Y | N | Y | Y | Y | N | Y | Y | Y | Y |
| [39] | [70] | Amoah PA et al. （2021） | Y | Y | N | Y | Y | CT | N | Y | Y | Y | Y |
| [40] | [71] | Chen W et al. (2020) | Y | Y | N | Y | Y | Y | N | Y | Y | Y | Y |
| [41] | [72] | Xu G et al. (2022) | Y | Y | Y | Y | Y | Y | N | Y | Y | Y | Y |
| [42] | [73] | Rivadeneira MF et al. (2022) | Y | Y | Y | Y | Y | Y | N | Y | Y | Y | Y |
| [43] | [74] | Choi S (2024) | Y | Y | Y | Y | Y | Y | N | Y | Y | Y | Y |
| [44] | [75] | Rivadeneira MF et al. (2022) | Y | Y | Y | Y | Y | Y | N | Y | Y | Y | Y |
| [45] | [76] | Ha L N et al. (2023) | Y | Y | N | Y | Y | Y | N | Y | Y | Y | Y |
| [46] | [77] | Biscaldi V et al. (2023) | Y | Y | N | Y | Y | N | N | Y | Y | Y | Y |
| [47] | [78] | Reitegger F et al. (2023) | Y | Y | N | Y | Y | Y | N | Y | Y | Y | Y |
| [48] | [79] | Xie C Y et al. (2020) | Y | Y | N | Y | Y | Y | N | Y | Y | Y | Y |
| [49] | [80] | Tran HTT et al. (2022) | Y | Y | N | Y | Y | Y | N | Y | Y | Y | Y |
| [50] | [81] | Wang Y et al. (2022) | Y | Y | Y | Y | Y | Y | N | Y | Y | Y | Y |
| [51] | [82] | Ryan Michael F et al. (2021) | Y | Y | Y | Y | Y | Y | N | Y | Y | Y | Y |
| [52] | [83] | VÂJÂEan CC et al. (2015) | Y | Y | N | Y | Y | Y | N | Y | Y | Y | Y |
| [53] | [84] | Amoako I et al. (2023) | Y | Y | N | Y | Y | Y | N | Y | Y | Y | Y |
| [54] | [85] | Kim S et al. (2021) | Y | Y | N | Y | Y | Y | N | Y | Y | Y | Y |
| [55] | [86] | Paige SR et al.（2017） | Y | Y | N | Y | Y | Y | Y | Y | Y | Y | Y |
| [56] | [87] | Zhong M et al.（2016） | Y | Y | N | Y | Y | Y | N | Y | Y | Y | Y |
| [57] | [88] | Hu J M et al. (2022) | Y | Y | N | Y | Y | Y | N | Y | Y | Y | Y |
| [58] | [89] | Yu Y et al. (2019) | Y | Y | N | Y | Y | Y | N | Y | Y | Y | Y |
| [59] | [90] | Tong W et al. (2023) | Y | Y | N | Y | Y | Y | N | Y | Y | Y | Y |
| [60] | [91] | Luo YF et al. (2018) | Y | Y | Y | Y | Y | CT | N | Y | Y | Y | Y |
| [61] | [92] | Jiang L H et al. (2022) | Y | Y | N | Y | Y | Y | N | Y | Y | Y | Y |
| [62] | [93] | Park J W et al. （2017） | Y | Y | Y | Y | Y | Y | Y | Y | Y | Y | Y |
| [63] | [94] | Hsu W et al. (2014) | Y | Y | Y | Y | Y | Y | N | Y | Y | Y | Y |
| [64] | [95] | Ju-Young H et al. (2019) | Y | Y | Y | Y | Y | Y | N | Y | Y | Y | Y |
| [65] | [96] | Hwang A R et al. (2019) | Y | Y | Y | Y | Y | Y | N | Y | Y | Y | Y |
| [66] | [97] | Kim KA et al.（2023） | Y | Y | Y | Y | Y | Y | N | Y | Y | Y | Y |
| [67] | [98] | Li S J et al. (2022) | Y | Y | N | Y | Y | Y | N | Y | Y | Y | Y |
| [68] | [99] | Cui G H et al. (2020) | Y | Y | N | Y | Y | Y | N | Y | Y | Y | Y |
| [69] | [100] | Wu Q et al. (2022) | Y | Y | N | Y | Y | Y | N | Y | Y | Y | Y |
| [70] | [101] | Kasımoğlu N et al. (2023) | Y | Y | N | Y | Y | Y | N | Y | Y | Y | Y |
| [71] | [102] | Eyimaya A et al. (2021) | Y | Y | N | Y | Y | Y | N | Y | Y | Y | Y |
| [72] | [103] | Wang S S et al. (2015) | Y | Y | N | Y | Y | Y | N | Y | Y | Y | Y |
| [73] | [104] | Lee S M et al. (2018) | Y | Y |  | Y | Y |  |  | Y | Y | Y | Y |
| [74] | [105] | Öztürk E et al. (2023) | Y | Y | Y | Y | Y | Y | N | Y | Y | Y | Y |
| [75] | [106] | Meng S X et al. （2018） | Y | Y | N | Y | Y | Y | N | Y | Y | Y | Y |
| [76] | [107] | Tian H et al. (2022) | Y | Y | N | Y | Y | Y | N | Y | Y | Y | Y |
| [77] | [108] | [Tariq A et al. (2020](https://pubmed.ncbi.nlm.nih.gov/?term=Tariq A[Author])) | Y | Y | N | Y | Y | Y | N | Y | Y | Y | Y |
| [78] | [109] | Huang CL et al. (2020) | Y | Y | N | Y | Y | Y | Y | Y | Y | Y | Y |
| [79] | [110] | Lee B C et al. (2021) | Y | Y | N | Y | Y | Y | N | Y | Y | Y | Y |
| [80] | [111] | Yang SC et al. (2019) | Y | Y | N | Y | Y | Y | N | Y | Y | Y | Y |
| [81] | [112] | Acar AK et al. (2021) | Y | Y | Y | Y | Y | N | N | Y | Y | Y | Y |
| [82] | [113] | Williams MSZ et al. （2014） | Y | Y | N | Y | Y | Y | N | Y | Y | Y | Y |
| [83] | [114] | Hong KJ et al. (2021) | Y | Y | Y | Y | Y | Y | N | Y | Y | Y | Y |
| [84] | [115] | Hadley MK( 2022) | Y | Y | N | Y | Y | Y | Y | Y | Y | Y | Y |
| [85] | [116] | Jiang X X et al. (2023) | Y | Y | N | Y | Y | Y | N | Y | Y | Y | Y |
| [86] | [117] | Liu J C et al. (2020) | Y | Y | N | Y | Y | Y | N | Y | Y | Y | Y |
| [87] | [118] | Luo L et al. (2021) | Y | Y | N | Y | Y | Y | N | Y | Y | Y | Y |
| [88] | [119] | Göde A et al. (2023) | Y | Y | Y | Y | Y | Y | N | Y | Y | Y | Y |
| [89] | [120] | Kaynak S et al. (2022) | Y | Y | N | Y | Y | Y | N | Y | Y | Y | Y |

Y: yes (score=1); N: no(score=0); CT: cannot tell(score=0)

| Citation in this file |  | Author(Year) | Results | | | | | Discussion | | Other | | Total score |  |
| --- | --- | --- | --- | --- | --- | --- | --- | --- | --- | --- | --- | --- | --- |
|  | Citation corresponding to the manuscript reference |  | 12.Were the basic data adequately described? | 13.Does the response rate raise concerns about non-response bias? | 14.If appropriate, was information about non-responders described? | 15.Were the results internally consistent? | 16.Were the results for the analyses described in the methods, presented? | 17.Were the authors’ discussions and conclusions justified by the results? | 18.Were the limitations of the study discussed? | 19.Were there any funding sources or conflicts of interest that may affect the authors’ interpretation of the results? | 20.Was ethical approval or consent of participants attained? |  | Study quality |
| [1] | [19] | Yuan T, et al. (2020) | Y | Y | N | Y | Y | Y | Y | N | Y | 16 | High |
| [2] | [20] | Yang SC et al. (2017) | Y | Y | N | Y | Y | Y | Y | N | Y | 17 | High |
| [3] | [21] | Patil U et al. (2021) | Y | CT | N | Y | Y | Y | Y | N | Y | 18 | High |
| [4] | [26] | Tsukahara S et al. (2020) | Y | Y | N | Y | Y | Y | Y | N | Y | 16 | High |
| [5] | [28] | Lotto M et al. (2023) | Y | N | N | Y | Y | Y | Y | CT | Y | 16 | High |
| [6] | [29] | [Ana Luiza Dallora et al. (2024](https://pubmed.ncbi.nlm.nih.gov/?term=Dallora AL[Author])) | Y | Y | N | Y | Y | Y | Y | N | Y | 17 | High |
| [7] | [38] | Oducado et al. (2020) | Y | Y | N | Y | Y | Y | Y | N | Y | 17 | High |
| [8] | [39] | Qin N et al. (2021) | Y | N | N | Y | Y | Y | Y | N | Y | 17 | High |
| [9] | [40] | Li S et al. (2021) | Y | N | N | Y | Y | Y | Y | N | Y | 17 | High |
| [10] | [41] | Qin N et al. (2021) | Y | N | N | Y | Y | Y | Y | N | Y | 17 | High |
| [11] | [42] | Mai, J. R et al. （2022） | Y | N | N | Y | Y | Y | Y | CT | Y | 16 | High |
| [12] | [43] | Aslantekin-Özcoban F et al. (2021) | N | N | N | Y | Y | Y | Y | N | Y | 17 | High |
| [13] | [44] | Kılınç İşleyen E et al. (2024) | Y | N | N | Y | Y | Y | N | N | Y | 15 | Moderate |
| [14] | [45] | Zhang S et al. (2024) | Y | N | N | Y | Y | Y | Y | N | Y | 18 | High |
| [15] | [46] | Mayukh NJJoC (2024) | N | N | N | Y | Y | Y | Y | CT | N | 14 | Moderate |
| [16] | [47] | Bao, X. L et al. (2022) | Y | N | N | Y | Y | Y | Y | CT | Y | 16 | High |
| [17] | [48] | Sögüt S et al. (2022) | Y | N | N | Y | Y | Y | Y | N | Y | 17 | High |
| [18] | [49] | Sun H(2022) | Y | N | N | Y | Y | Y | Y | N | Y | 18 | High |
| [19] | [50] | Turan N et al. (2021) | N | N | N | Y | Y | Y | Y | N | Y | 16 | High |
| [20] | [51] | Pisl V et al. (2021) | N | N | N | Y | Y | Y | Y | N | Y | 14 | Moderate |
| [21] | [52] | Zadeh Kh et al. (2023) | Y | N | N | Y | Y | Y | N | N | Y | 17 | High |
| [22] | [53] | Chen, Y. N et al. （2023） | N | N | N | Y | Y | Y | Y | CT | Y | 15 | Moderate |
| [23] | [54] | Chun, H. R et al. (2021) | N | N | N | Y | Y | Y | Y | CT | Y | 15 | Moderate |
| [24] | [55] | Pisl V et al. (2021) | N | N | N | Y | Y | Y | Y | N | Y | 14 | Moderate |
| [25] | [56] | Kıbrıs Ş et al. (2023) | N | N | N | Y | Y | Y | Y | N | Y | 17 | High |
| [26] | [57] | Liao LL et al. (2024) | Y | N | N | Y | Y | Y | Y | N | Y | 18 | High |
| [27] | [58] | Fehér A et al. (2021) | Y | Y | N | Y | Y | Y | Y | N | Y | 15 | Moderate |
| [28] | [59] | Noh M Y (2021) | Y | N | N | Y | Y | Y | N | CT | Y | 15 | Moderate |
| [29] | [60] | Britt RK et al. (2017) | N | N | N | Y | Y | Y | Y | N | Y | 16 | High |
| [30] | [61] | Yan X D et al. （2018） | Y | Y | N | Y | Y | Y | Y | CT | Y | 15 | Moderate |
| [31] | [62] | Wang X et al. (2022) | Y | N | N | Y | Y | Y | Y | N | Y | 17 | High |
| [32] | [63] | Chen SC et al. （2022） | Y | N | N | Y | Y | Y | Y | N | Y | 17 | High |
| [33] | [64] | Chen SC et al. (2023) | Y | N | N | Y | Y | Y | Y | N | Y | 17 | High |
| [34] | [65] | Kim J O (2017) | Y | N | N | Y | Y | Y | Y | CT | N | 16 | High |
| [35] | [66] | Nam Y H et al. (2020) | Y | N | N | Y | Y | Y | Y | CT | Y | 16 | High |
| [36] | [67] | Masilamani V et al. （2020） | N | N | N | Y | Y | Y | Y | CT | N | 14 | Moderate |
| [37] | [68] | Kim H S et al. (2021) | Y | N | N | Y | Y | Y | N | CT | Y | 16 | High |
| [38] | [69] | Kuang H D et al. （2023） | Y | N | N | Y | Y | Y | Y | CT | Y | 16 | High |
| [39] | [70] | Amoah PA et al. （2021） | N | N | N | Y | Y | Y | N | N | Y | 14 | Moderate |
| [40] | [71] | Chen W et al. (2020) | Y | N | N | Y | Y | Y | Y | N | Y | 17 | High |
| [41] | [72] | Xu G et al. (2022) | Y | N | N | Y | Y | Y | Y | N | Y | 18 | High |
| [42] | [73] | Rivadeneira MF et al. (2022) | Y | N | N | Y | Y | Y | Y | N | Y | 18 | High |
| [43] | [74] | Choi S (2024) | Y | N | N | Y | Y | Y | Y | N | Y | 18 | High |
| [44] | [75] | Rivadeneira MF et al. (2022) | Y | N | N | Y | Y | Y | Y | N | Y | 18 | High |
| [45] | [76] | Ha L N et al. (2023) | Y | N | N | Y | Y | Y | N | CT | Y | 15 | Moderate |
| [46] | [77] | Biscaldi V et al. (2023) | N | N | Y | Y | Y | Y | Y | CT | Y | 15 | Moderate |
| [47] | [78] | Reitegger F et al. (2023) | Y | N | N | Y | Y | Y | N | N | Y | 16 | High |
| [48] | [79] | Xie C Y et al. (2020) | Y | N | N | Y | Y | Y | Y | CT | Y | 16 | High |
| [49] | [80] | Tran HTT et al. (2022) | Y | Y | N | Y | Y | Y | Y | N | Y | 16 | High |
| [50] | [81] | Wang Y et al. (2022) | Y | N | N | Y | Y | Y | Y | CT | Y | 17 | High |
| [51] | [82] | Ryan Michael F et al. (2021) | Y | N | N | Y | Y | Y | Y | CT | Y | 17 | High |
| [52] | [83] | VÂJÂEan CC et al. (2015) | Y | N | N | Y | Y | Y | Y | CT | Y | 16 | High |
| [53] | [84] | Amoako I et al. (2023) | Y | Y | N | Y | Y | Y | Y | N | Y | 16 | High |
| [54] | [85] | Kim S et al. (2021) | Y | N | N | Y | Y | Y | Y | N | Y | 17 | High |
| [55] | [86] | Paige SR et al.（2017） | Y | Y | N | Y | Y | Y | Y | N | Y | 17 | High |
| [56] | [87] | Zhong M et al.（2016） | Y | N | N | Y | Y | Y | N | CT | Y | 15 | Moderate |
| [57] | [88] | Hu J M et al. (2022) | Y | N | N | Y | Y | Y | Y | CT | Y | 16 | High |
| [58] | [89] | Yu Y et al. (2019) | Y | N | N | Y | Y | Y | Y | N | Y | 17 | High |
| [59] | [90] | Tong W et al. (2023) | N | N | N | Y | Y | Y | Y | N | Y | 16 | High |
| [60] | [91] | Luo YF et al. (2018) | N | N | N | Y | Y | Y | Y | N | Y | 16 | High |
| [61] | [92] | Jiang L H et al. (2022) | Y | N | N | Y | Y | Y | Y | N | Y | 17 | High |
| [62] | [93] | Park J W et al. （2017） | Y | N | N | Y | Y | Y | Y | CT | Y | 18 | High |
| [63] | [94] | Hsu W et al. (2014) | N | N | N | Y | Y | Y | Y | N | Y | 17 | High |
| [64] | [95] | Ju-Young H et al. (2019) | N | N | N | Y | Y | Y | N | CT | Y | 15 | Moderate |
| [65] | [96] | Hwang A R et al. (2019) | Y | N | N | Y | Y | Y | Y | N | Y | 18 | High |
| [66] | [97] | Kim KA et al.（2023） | Y | Y | N | Y | Y | Y | Y | N | Y | 17 | High |
| [67] | [98] | Li S J et al. (2022) | Y | N | N | Y | Y | Y | Y | CT | Y | 16 | High |
| [68] | [99] | Cui G H et al. (2020) | Y | N | N | Y | Y | Y | Y | CT | Y | 16 | High |
| [69] | [100] | Wu Q et al. (2022) | Y | N | N | Y | Y | Y | N | CT | Y | 15 | Moderate |
| [70] | [101] | Kasımoğlu N et al. (2023) | Y | N | N | Y | Y | Y | Y | N | Y | 17 | High |
| [71] | [102] | Eyimaya A et al. (2021) | N | Y | N | Y | Y | Y | N | CT | Y | 13 | Moderate |
| [72] | [103] | Wang S S et al. (2015) | Y | N | N | Y | Y | Y | Y | CT | Y | 16 | High |
| [73] | [104] | Lee S M et al. (2018) |  |  |  | Y | Y | Y | Y | N | Y | 14 | Moderate |
| [74] | [105] | Öztürk E et al. (2023) | Y | N | N | Y | Y | Y | Y | N | Y | 18 | High |
| [75] | [106] | Meng S X et al. （2018） | Y | N | N | Y | Y | Y | N | CT | Y | 15 | Moderate |
| [76] | [107] | Tian H et al. (2022) | Y | N | N | Y | Y | Y | Y | N | Y | 17 | High |
| [77] | [108] | [Tariq A et al. (2020](https://pubmed.ncbi.nlm.nih.gov/?term=Tariq A[Author])) | Y | Y | N | Y | Y | Y | Y | N | Y | 16 | High |
| [78] | [109] | Huang CL et al. (2020) | N | N | N | Y | Y | Y | Y | N | Y | 17 | High |
| [79] | [110] | Lee B C et al. (2021) | Y | N | N | Y | Y | Y | Y | CT | N | 15 | Moderate |
| [80] | [111] | Yang SC et al. (2019) | N | N | N | Y | Y | Y | Y | N | Y | 16 | High |
| [81] | [112] | Acar AK et al. (2021) | Y | N | N | Y | Y | Y | Y | N | Y | 17 | High |
| [82] | [113] | Williams MSZ et al. （2014） | Y | N | N | Y | Y | Y | Y | CT | Y | 16 | High |
| [83] | [114] | Hong KJ et al. (2021) | Y | N | N | Y | Y | Y | Y | N | Y | 18 | High |
| [84] | [115] | Hadley MK( 2022) | N | N | N | Y | Y | Y | Y | CT | Y | 16 | High |
| [85] | [116] | Jiang X X et al. (2023) | Y | N | N | Y | Y | Y | Y | CT | Y | 16 | High |
| [86] | [117] | Liu J C et al. (2020) | Y | N | N | Y | Y | Y | N | CT | Y | 15 | Moderate |
| [87] | [118] | Luo L et al. (2021) | Y | N | N | Y | Y | Y | Y | N | Y | 17 | High |
| [88] | [119] | Göde A et al. (2023) | Y | N | N | Y | Y | Y | N | N | Y | 17 | High |
| [89] | [120] | Kaynak S et al. (2022) | Y | Y | N | Y | Y | Y | Y | N | Y | 16 | High |

Y: yes (score=1); N: no(score=0); CT: cannot tell(score=0)

**References**

1. Yuan T, Liu H, Li XD, Liu HR: **Factors Affecting Infection Control Behaviors to Prevent COVID-19: An Online Survey of Nursing Students in Anhui, China in March and April 2020**. *Medical science monitor : international medical journal of experimental and clinical research* 2020, **26**:e925877.

2. Yang SC, Luo YF, Chiang CH: **The Associations Among Individual Factors, eHealth Literacy, and Health-Promoting Lifestyles Among College Students**. *Journal of medical Internet research* 2017, **19**(1):e15.

3. Patil U, Kostareva U, Hadley M, Manganello JA, Okan O, Dadaczynski K, Massey PM, Agner J, Sentell T: **Health Literacy, Digital Health Literacy, and COVID-19 Pandemic Attitudes and Behaviors in U.S. College Students: Implications for Interventions**. *International journal of environmental research and public health* 2021, **18**(6).

4. Tsukahara S, Yamaguchi S, Igarashi F, Uruma R, Ikuina N, Iwakura K, Koizumi K, Sato Y: **Association of eHealth Literacy With Lifestyle Behaviors in University Students: Questionnaire-Based Cross-Sectional Study**. *Journal of medical Internet research* 2020, **22**(6):e18155.

5. Lotto M, Maschio KF, Silva KK, Ayala Aguirre PE, Cruvinel A, Cruvinel T: **eHEALS as a predictive factor of digital health information seeking behavior among Brazilian undergraduate students**. *Health Promot Int* 2023, **38**(4).

6. Dallora AL, Andersson EK, Gregory Palm B, Bohman D, Björling G, Marcinowicz L, Stjernberg L, Anderberg P: **Nursing Students' Attitudes Toward Technology: Multicenter Cross-Sectional Study**. *JMIR Med Educ* 2024, **10**:e50297.

7. Oducado RMJAoTM, Health P: **Filipino nursing students' ehealth literacy and criteria used for selection of health websites**. 2020, **23**.

8. Qin N, Shi S, Ma G, Li X, Duan Y, Shen Z, Luo A, Zhong Z: **Associations of COVID-19 Risk Perception, eHealth Literacy, and Protective Behaviors Among Chinese College Students Following Vaccination: A Cross-Sectional Study**. *Frontiers in public health* 2021, **9**:776829.

9. Li S, Cui G, Kaminga AC, Cheng S, Xu H: **Associations Between Health Literacy, eHealth Literacy, and COVID-19-Related Health Behaviors Among Chinese College Students: Cross-sectional Online Study**. *Journal of medical Internet research* 2021, **23**(5):e25600.

10. Qin N, Shi S, Duan Y, Ma G, Li X, Shen Z, Zhang S, Luo A, Zhong Z: **Social Media Use, eHealth Literacy, Knowledge, Attitudes, and Practices Toward COVID-19 Vaccination Among Chinese College Students in the Phase of Regular Epidemic Prevention and Control: A Cross-Sectional Survey**. *Frontiers in public health* 2021, **9**:754904.

11. Mai, J. R., Zhou, L., He, J. N., Huang, T. F., & Lin, L. N. Correlative Analysis of E-Health Literacy and Infectious Disease Health Literacy Among Nursing Undergraduates in Guangdong Province [J]. Chinese Nursing Education, 2022, 19(08): 719-722. DOI: 10.3761/j.issn.1672-9234.2022.08.010

12. Aslantekin-Özcoban F, Gün MJC, Obstetrics E, Gynecology: **Emergency contraception knowledge level and e-health literacy in Turkish university students**. 2021, **48**(6):1424-1431.

13. Kılınç İşleyen E, Korkmaz Aslan G, Kartal A: **Knowledge and Perceptions About Cervical Cancer and Human Papillomavirus, and Relationship with E-health Literacy, and Affecting Factors Among Female University Students**. *Journal of adolescent and young adult oncology* 2024, **13**(3):564-572.

14. Zhang S, Wang W, Wu S, Ye H, Dong L, Wang J, Ning X, Cui H: **Analysis of the mediating effect between ehealth literacy and health self-management of undergraduate nursing students' mental health literacy**. *BMC nursing* 2024, **23**(1):264.

15. Mayukh NJJoC, Language, Culture: **The Influence of eHealth Literacy and Self-Efficacy on Online Health Information-Seeking Behaviour among University Students: Cyberchondria as a Mediator**. 2024, **4**(1):40-60.

16. Bao, X. L. Research on the influence of college students' epidemic prevention and control cognition on their healthy lifestyle [Doctoral dissertation, Southern Medical University]. 2022. DOI: 10.27003/d.cnki.gojyu.2022.001045

17. Sögüt S, Cangöl E, Dolu İ: **The Relationship Between eHealth Literacy and Self-Efficacy Levels in Midwifery Students Receiving Distance Education During the COVID-19 Pandemic**. *J Nurs Res* 2022, **30**(2):e203.

18. Sun H, Qian L, Xue M, Zhou T, Qu J, Zhou J, Qu J, Ji S, Bu Y, Hu Y *et al*: **The relationship between eHealth literacy, social media self-efficacy and health communication intention among Chinese nursing undergraduates: A cross-sectional study**. *Frontiers in public health* 2022, **10**:1030887.

19. Turan N, Güven Özdemir N, Çulha Y, Özdemir Aydın G, Kaya H, Aştı T: **The effect of undergraduate nursing students' e-Health literacy on healthy lifestyle behaviour**. *Glob Health Promot* 2021, **28**(3):6-13.

20. Pisl V, Volavka J, Chvojkova E, Cechova K, Kavalirova G, Vevera J: **Dissociation, Cognitive Reflection and Health Literacy Have a Modest Effect on Belief in Conspiracy Theories about COVID-19**. *International journal of environmental research and public health* 2021, **18**(10).

21. Zadeh Kh., Ghazavi, Salamat A. J. Health Literacy: Investigating the Relationship Between Health Literacy, Conspiracy Beliefs, and Future Anxiety in the Face of COVID-19. Journal Name, 2023, 20(1), 50–55. DOI: 10.48305/him.2023.41589.1099

22. Chen, Y. N. A study on cervical cancer information-seeking behavior among female college students [Master’s thesis, Sichuan International Studies University]. 2023.DOI: 10.27348/d.cnki.gscwc.2023.000269.

23. Chun, H. R., Yoon, H. R., & Choi, S. G., Korean Journal of Population Studies: Digital health literacy and preventive health behaviors among college students: Focusing on COVID-19 vaccination intention and participation in preventive measures. 2021, 44(2):121-141.DOI: 10.31693/KJPS.2021.06.44.2.121

24. Pisl V, Volavka J, Chvojkova E, Cechova K, Kavalirova G, Vevera J: **Willingness to Vaccinate Against COVID-19: The Role of Health Locus of Control and Conspiracy Theories**. *Frontiers in psychology* 2021, **12**:717960.

25. Kıbrıs Ş, Kızılkaya SJSvSRAD: **E-SAĞLIK OKURYAZARLIK DÜZEYİNİN SAĞLIK ALGISI ÜZERİNE ETKİSİNİN İNCELENMESİ**. 2023, **5**(2):241-250.

26. Liao LL, Chang LC, Lai IJ, Lee CK: **College Students' E-health Literacy, Social Media Use, and Perceptions of E-cigarettes in Taiwan**. *J Community Health* 2024, **49**(1):52-60.

27. Fehér A, Véha M, Boros HM, Kovács B, Kontor E, Szakály Z: **The Relationship between Online and Offline Information-Seeking Behaviors for Healthy Nutrition**. *International journal of environmental research and public health* 2021, **18**(19).

28. Noh M Y. The effect of e-health literacy on exercise self-schema among female college students in vocational schools. Journal of the Korean Association of Physical Education and Sport for Girls and Women, 2021, 35(1), 85-97.DOI : 10.16915/jkapesgw.2021.3.35.1.85

29. Britt RK, Collins WB, Wilson K, Linnemeier G, Englebert AM: **eHealth Literacy and Health Behaviors Affecting Modern College Students: A Pilot Study of Issues Identified by the American College Health Association**. *Journal of medical Internet research* 2017, **19**(12):e392.

30. Yan X D. Exploring the Mechanism of Effectively Using Mobile Healthcare Applications [D]. Tianjin University, 2018.

31. Wang X, Yue T, Mo PK: **The associations among cognitive social factors, eHealth literacy and health-promoting behaviors in Chinese adolescents**. *Health Promot Int* 2022, **37**(6).

32. Chen SC, Huy LD, Lin CY, Lai CF, Nguyen NTH, Hoang NY, Nguyen TTP, Dang LT, Truong NLT, Phan TN *et al*: **Association of Digital Health Literacy with Future Anxiety as Mediated by Information Satisfaction and Fear of COVID-19: A Pathway Analysis among Taiwanese Students**. *International journal of environmental research and public health* 2022, **19**(23).

33. Chen SC, Hong Nguyen NT, Lin CY, Huy LD, Lai CF, Dang LT, Truong NLT, Hoang NY, Nguyen TTP, Phaṇ TN *et al*: **Digital health literacy and well-being among university students: Mediating roles of fear of COVID-19, information satisfaction, and internet information search**. *Digital health* 2023, **9**:20552076231165970.

34. Kim J O. The Reliability of Health Information on the Internet and the Medical Advertising’s Attitude on the Internet according to e-Health Literacy Level. Humanities and Social Sciences, 2017, 8(4):299-314.

35. Nam Y H. A comparative study of e-health literacy, health information credibility, and health behaviors affecting health information usage motivation between Korean and Chinese university students. Journal of Digital Contents Society, 2020, 21(3):513-520. DOI : 10.9728/dcs.2020.21.3.513

36. Masilamani V, Arulchelvan S, Rozario AMJCRCdCyE: **Alfabetización en e-Salud de los jóvenes: Credibilidad y calidad de la información sanitaria con móviles en la India**. 2020(64):85-95.

37. Kim H S, Lee K H. A study on perceptions and activation strategies of community service activities among health sciences university students. The Journal of the Korea Academia-Industrial Cooperation Society, 2021, 22(10):304-315. DOI : 10.5762/KAIS.2021.22.10.304

38. Kuang H D, Li J, Gu Z J, et al. The mediating effect of e-health literacy between mental health and online psychological help-seeking behavior among college students [J]. China Journal of Health Psychology, 2023, 31(12): 1876-1880. DOI:10.13342/j.cnki.cjhp.2023.12.022.

39. Amoah PA, Leung AYM, Parial LL, Poon ACY, Tong HH, Ng WI, Li X, Wong EML, Kor PPK, Molassiotis A: **Digital Health Literacy and Health-Related Well-Being Amid the COVID-19 Pandemic: The Role of Socioeconomic Status Among University Students in Hong Kong and Macao**. *Asia-Pacific journal of public health* 2021, **33**(5):613-616.

40. Chen W, Zheng Q, Liang C, Xie Y, Gu D: **Factors Influencing College Students' Mental Health Promotion: The Mediating Effect of Online Mental Health Information Seeking**. *International journal of environmental research and public health* 2020, **17**(13).

41. Xu G, Xu Y, Tu X, Hao S, Liu T: **The Association between Self-Rated Health and Health Self-Management Ability of Healthcare Undergraduates: The Chain Mediating Roles of eHealth Literacy and Resistance to Peer Influence**. *International journal of environmental research and public health* 2022, **19**(21).

42. Rivadeneira MF, Miranda-Velasco MJ, Arroyo HV, Caicedo-Gallardo JD, Salvador-Pinos C: **Digital Health Literacy Related to COVID-19: Validation and Implementation of a Questionnaire in Hispanic University Students**. *International journal of environmental research and public health* 2022, **19**(7).

43. Choi S: **Comparison of Self-Tracking Health Practices, eHealth Literacy, and Subjective Well-Being Between College Students With and Without Disabilities: Cross-Sectional Survey**. *JMIR formative research* 2024, **8**:e48783.

44. Rivadeneira MF, Salvador C, Araujo L, Caicedo-Gallardo JD, Cóndor J, Torres-Castillo AL, Miranda-Velasco MJ, Dadaczynski K, Okan O: **Digital health literacy and subjective wellbeing in the context of COVID-19: A cross-sectional study among university students in Ecuador**. *Frontiers in public health* 2022, **10**:1052423.

45. Ha L N, Chang Q N, Chen X. The impact of e-health literacy on well-being in medical students: A serial mediation model of basic psychological needs and negative emotions [J]. China Journal of Health Psychology, 2023, 31(9): 1381-1388. DOI: 10.13342/j.cnki.cjhp.2023.09.019

46. Biscaldi V, Delbosq S, Ghelfi M, Serio J, Vecchio L, Dadaczynski K, Okan O, Velasco V: **A cross-sectional study of university students' wellbeing: What to focus on?** *PSICOLOGIA DELLA SALUTE* 2023:105-124.

47. Reitegger F, Wright M, Berger J, Gasteiger-Klicpera B: **Digitale Gesundheitskompetenz und Wohlbefinden**. *Prävention und Gesundheitsförderung* 2023, **18**(2):204-210.

48. Xie C Y, Li S J, Hu J Y. Association between e-health literacy, social support and depressive symptoms among female nursing students [J]. Chinese Journal of School Health, 2020, 41(5): 716-719. DOI: 10.16835/j.cnki.1000-9817.2020.05.022.

49. Tran HTT, Nguyen MH, Pham TTM, Kim GB, Nguyen HT, Nguyen NM, Dam HTB, Duong TH, Nguyen YH, Do TT *et al*: **Predictors of eHealth Literacy and Its Associations with Preventive Behaviors, Fear of COVID-19, Anxiety, and Depression among Undergraduate Nursing Students: A Cross-Sectional Survey**. *International journal of environmental research and public health* 2022, **19**(7).

50. Wang Y. The influence of e-health literacy and health anxiety on cyberchondria among university students [D]. Yanbian University, 2022. DOI: 10.27439/d.cnki.gybdu.2022.000603.

51. Ryan Michael F, Tuppal CP, Estoque HV, Sadang JM, Superio DL, Don Vicente C, Mary Nellie T, Xerxes G, Quiros JD, Fajardo MTR: **Uso de Internet y la alfabetización en eSalud con temor al COVID-19 entre estudiantes de enfermería en Filipinas Internet use, eHealth literacy and fear of COVID-19 among nursing students in the Philippines**.

52. VÂJÂEan CC, BĂBan A: **EMOTIONAL AND BEHAVIORAL CONSEQUENCES OF ONLINE HEALTH INFORMATION-SEEKING: THE ROLE OF EHEALTH LITERACY**. *Cognitie, Creier, Comportament/Cognition, Brain, Behavior* 2015, **19**(4):327-345.

53. Amoako I, Srem-Sai M, Quansah F, Anin S, Agormedah EK, Hagan Jnr JE: **Moderation modelling of COVID-19 digital health literacy and sense of coherence across subjective social class and age among university students in Ghana**. *BMC Psychol* 2023, **11**(1):337.

54. Kim S, Oh J: **The Relationship between E-Health Literacy and Health-Promoting Behaviors in Nursing Students: A Multiple Mediation Model**. *Int J Environ Res Public Health* 2021, **18**(11).

55. Paige SR, Stellefson M, Chaney BH, Chaney JD, Alber JM, Chappell C, Barry AE: **Examining the Relationship between Online Social Capital and eHealth Literacy: Implications for Instagram Use for Chronic Disease Prevention among College Students**. *Am J Health Educ* 2017, **48**(4):264-277.

56. , Xu X Y. Association between individual factors, e-health literacy and health information utilization among university students in Guangzhou [J]. Chinese Journal of School Health, 2016, 37(12): 1787-1790. DOI: 10.16835/j.cnki.1000-9817.2016.12.009

57. Hu J M, Li H L, Yang Y L, Zhang Y W, He X F, Shi L. Investigation of college students' ability to identify online rumors during public health emergencies [J]. Journal of Nursing Science, 2022, 37(8): 65-68+93. DOI: 10.3870/j.issn.1001-4152.2022.08.065

58. Yu Y, Yan X, Zhang X, Zhou S: **What they gain depends on what they do: an exploratory empirical research on effective use of mobile healthcare applications**. 2019.

59. Tong W, Meng S: **Effects of Physical Activity on Mobile Phone Addiction Among College Students: The Chain-Based Mediating Role of Negative Emotion and E-Health Literacy**. *Psychol Res Behav Manag* 2023, **16**:3647-3657.

60. Luo YF, Yang SC, Chen AS, Chiang CH: **Associations of eHealth Literacy With Health Services Utilization Among College Students: Cross-Sectional Study**. *Journal of medical Internet research* 2018, **20**(10):e283.

61. Jiang L H, Guo X Y, Lu B Y, et al. Correlation between e-health literacy and physical health among college students [J]. Chinese Journal of School Health, 2022, 43(7): 990-994. DOI: 10.16835/j.cnki.1000-9817.2022.07.008

62. Park J W. A comparative study of e-health literacy and self-care competence between nursing students and non-health major female college students [J]. Journal of Korean Academy of Nursing Administration, 2017, 23(4): 439-449. DOI: 10.11111/jkana.2017.23.4.439.

63. Hsu W, Chiang C, Yang S: **The effect of individual factors on health behaviors among college students: the mediating effects of eHealth literacy**. *Journal of medical Internet research* 2014, **16**(12):e287.

64. Ju-Young H, Lee S-YJM-LU: **The relationship between the subjective health status, e-Health literacy, health literacy and health promoting behavior in under graduate nursing students**. 2019, **19**(1).

65. Hwang A R, Je J J. The influence of university students' e-health literacy on health promotion behaviors[J]. 2019, 32(3). DOI: 10.15434/kssh.2019.32.3.165

66. Kim KA, Hyun MS, De Gagne JC, Ahn JA: **A cross-sectional study of nursing students' eHealth literacy and COVID-19 preventive behaviours**. *Nursing open* 2023, **10**(2):544-551.

67. Li S J, Cui G H, Xu H L. Path analysis of internet social support, e-health literacy and health-related behaviors among college students [J]. Chinese Journal of Health Statistics, 2022, 39(1): 118-121. DOI: 10.3969/j.issn.1002-3674.2022.01.027.

68. Cui G H, Yin Y T, Wang M Z, et al. The relationship between e-health literacy and healthy lifestyles among medical students [J]. Chinese Journal of School Health, 2020, 41(6): 936-938. DOI: 10.16835/j.cnki.1000-9817.2020.06.037.

69. Wu Q, Zhao G H, Gong J, et al. Status and correlation analysis of e-health literacy and healthy lifestyles among university students in Wuhan [J]. Medicine and Society, 2022, 35(8): 78-83. DOI: 10.13723/j.yxysh.2022.08.015.

70. Kasımoğlu N, Karakurt P, Başkan SAJIJOHSR, Policy: **THE RELATIONSHIP BETWEEN UNIVERSITY STUDENTS’E-HEALTH LITERACY AND HEALTHY LIFESTYLE BEHAVIORS**. 2023, **8**(1):38-47.

71. Eyimaya A, Özdemir F, Tezel A, Apay SE: **Determining the healthy lifestyle behaviors and e-health literacy levels in adolescents**. *Revista da Escola de Enfermagem da U S P* 2021, **55**:e03742.

72. WWang S S. Research on the eHealth literacy of college students in Hangzhou [D]. Hangzhou Normal University, 2015.

73. Lee S M. The Effect of e-Health literacy on Health Behavior in Health Science Majors [J]. The Journal of Korean Society for School & Community Health Education, 2018, 19(2): 77-86.

74. Öztürk E, Işık SS, Can ZJHSHD: **Determining the Relationship Between e-Health Literacy and Health-Improving and Protective Behaviors in Nursing Students**. **5**(2):106-116.

75. Meng S X, Shen C. Investigation on e-health literacy and behavior status among university students in Nanjing[J]. Chinese Journal of Health Education, 2018, 34(3): 254-257. DOI: 10.16168/j.cnki.issn.1002-9982.2018.03.014

76. Tian H, Chen J: **The association and intervention effect between eHealth literacy and lifestyle behaviors among Chinese university students**. *Revista da Escola de Enfermagem da U S P* 2022, **56**:e20220147.

77. Tariq A, Khan SR, Basharat A: **Internet Use, eHealth Literacy, and Dietary Supplement Use Among Young Adults in Pakistan: Cross-Sectional Study**. *Journal of medical Internet research* 2020, **22**(6):e17014.

78. Huang CL, Yang SC, Chiang CH: **The Associations between Individual Factors, eHealth Literacy, and Health Behaviors among College Students**. *International journal of environmental research and public health* 2020, **17**(6).

79. Lee B C. The relationship between e-health literacy and health behaviors among university students [J]. Journal of Convergence for Sport Science, 2021, 19(2): 55-62. DOI: 10.22997/jcses.2021.19.2.55.

80. Yang SC, Luo YF, Chiang CH: **Electronic Health Literacy and Dietary Behaviors in Taiwanese College Students: Cross-Sectional Study**. *Journal of medical Internet research* 2019, **21**(11):e13140.

81. Acar AK, Savcı S, Kahraman BÖ, Tanrıverdi AJJoB, Sciences CH: **Comparison of E-Health Literacy, Digital Health and Physical Activity Levels Of University Students In Different Fields**. 2021, **8**(2):380-389.

82. Williams MSZ: **A mixed methods study of health literacy and its role in hpv vaccine uptake among college students**; 2014.

83. Hong KJ, Park NL, Heo SY, Jung SH, Lee YB, Hwang JH: **Effect of e-Health Literacy on COVID-19 Infection-Preventive Behaviors of Undergraduate Students Majoring in Healthcare**. *Healthcare (Basel, Switzerland)* 2021, **9**(5).

84. Hadley MK: **COVID-19 and digital health literacy in university students/narrative competence and cognitive mapping as a culturally sustaining pedagogy in the education of emergent bilinguals**. 2022.

85. Jiang X X. Prevention Behavior and Influencing Factors of COVID-19 [D]. Shandong University, 2023.

86. Liu J C, Yin Y T, Fan Y Y. Relationship between eHealth literacy and illness behavior among vocational college students in Jinan City[J]. Chinese Journal of School Health, 2020, 41(10): 1502-1505+1510. DOI: 10.16835/j.cnki.1000-9817.2020.10.016.

87. Luo L, Song N Q, Yuan J F, et al. Relationship between electronic health literacy and dysmenorrhea management behavior of female college students in Guizhou universities [J]. Modern Preventive Medicine, 2021, 48(23):4317-4323,4330.

88. Göde A, Öztürk YE, Kuşcu FNJJoIHS, Management: **Examining The Relationship Between E-Health Literacy and Rational Drug Use: A Study on University Students**. 2023, **9**(18):8-16.

89. Kaynak S, Arat N, Yardımcı F, Şenol S, Yılmaz HBJEÜHFD: **Hemşirelik öğrencilerinin E-sağlık okuryazarlık düzeyi ile klinik karar verme becerileri arasındaki ilişki**. 2022, **38**(3):229-237.
